# Simultaneous Expression of Chicken Granulocyte Monocyte Colony-Stimulating Factor and the Hemagglutinin-Neuraminidase Epitope of the Virulent Newcastle Disease Virus Genotype VII C22 Strain in a Functional Synthetic Recombinant Adenovirus as a Genotype-Matched Vaccine with Potential Antiviral Activity

**DOI:** 10.1128/spectrum.04024-22

**Published:** 2023-04-10

**Authors:** Fathalrhman Eisa Addoma Adam, Xueliang Zhao, Zhao Guan, Zhengwu Chang, Michael Thrusfield, Kejia Lu, El Tigani Ahmed El Tigani-Asil, Abdelnasir Mohammed Adam Terab, Mohamedelfateh Ismael, Lina Tong, Daguia Wenam Prince-Theodore, Chen Luo, Sa Xiao, Xinglong Wang, Haijin Liu, Zengqi Yang

**Affiliations:** a College of Veterinary Medicine, Northwest A&F University, Yangling, China; b Veterinary Clinical Sciences Royal (Dick) School of Veterinary Studies, University of Edinburgh, Roslin, Midlothian, United Kingdom; c Veterinary Laboratories Division, Animal Wealth Sector, Abu Dhabi Agriculture and Food Safety Authority (ADAFSA), Abu Dhabi, United Arab Emirates; d Department of Preventive Medicine and Public Health, Faculty of Veterinary Science, University of Nyala, Nyala, Sudan; e College of Food Science and Engineering, Northwest Agriculture and Forestry University, Yangling, Shaanxi, China; University of Prince Edward Island

**Keywords:** adenovirus type 5, ChGM-CSF bio-adjuvant, chicken immunological genetic resistance, HN epitope of the genotype VII C22-strain, longer-term immunity, matched transgenic vaccine, Newcastle disease virus

## Abstract

When it comes to the prevention of clinical signs and mortality associated with infection of the Newcastle disease virus (NDV), vaccination has been very effective. However, recent evidence has proven that more highly virulent strains are emerging that bypass existing immune protection and pose a serious threat to the global poultry industry. Here, a novel rescued adenovirus 5-coexpressed chicken granulocyte monocyte colony-stimulating factor (ChGM-CSF) bio-adjuvant and C22-hemagglutinin-neuraminidase (HN) boosted chickens’ immunological genetic resistance and thus improved the immunological effectiveness of the critical new-generation vaccine *in vitro* and *in vivo*. Accordingly, the hemagglutination inhibition (HI) titers (log_2_) of the recombinant adenovirus (rAdv)-ChGM-CSF-HN-immunized chickens had greater, more persistent, and longer-lasting NDV-specific antibodies than the La Sota and rAdv-HN-inoculated birds. Moreover, humoral and adaptive immunological conditions were shown to be in harmony after rAdv-ChGM-CSF-HN inoculation and uniformly enhanced the expression of alpha interferon (IFN-α), IFN-β, IFN-γ, interleukin-1β (IL-1β), IL-2, IL-16, IL-18, and IL-22. Postchallenge, the control challenge (CC), wild-type adenovirus (wtAdv), and rAdv-ChGM-CSF groups developed unique NDV clinical manifestations, significant viral shedding, high tissue viral loads, gross and microscopic lesions, and 100% mortality within 7 days. The La Sota, rAdv-HN, and rAdv-ChGM-CSF-HN groups were healthy and had 100% survival rates. The rAdv-ChGM-CSF-HN group swiftly regulated and stopped viral shedding and had lower tissue viral loads than all groups at 5 days postchallenge (dpc). Thus, the antiviral activity of ChGM-CSF offered robust immune protection in the face of challenge and reduced viral replication convincingly. Our advance innovation concepts, combining ChGM-CSF with a field-circulating strain epitope, could lead to the development of a safe, genotype-matched, universal transgenic vaccine that could eradicate the disease globally, reducing poverty and food insecurity.

**IMPORTANCE** We studied the biological characterization of the developed functional synthetic recombinant adenoviruses, which showed a high degree of safety, thermostability, and genetic stability for up to 20 passages. It was demonstrated through both *in vitro* and *in vivo* testing that the immunogenicity of the proposed vaccine, which uses the T2A peptide from the Thosea asigna virus capsid protein supported by glycine and serine, helps with efficiency to generate a multicistronic vector, enables expression of two functional proteins in rAdv-ChGM-CSF-HN, and is superior to that of comparable vaccines. Additionally, adenovirus can be used to produce vaccines matching the virulent field-circulating strain epitope. Because there is no preexisting human adenoviral immunity detected in animals, the potency of adenoviral vaccines looks promising. Also, it ensures that the living vector does not carry the resistance gene that codes for the kanamycin antibiotic. Accordingly, a human recombinant adenoviral vaccine that has undergone biological improvements is beneficial and important.

## INTRODUCTION

Newcastle disease (ND), caused by the Newcastle disease virus (NDV), is a contagious, life-threatened transboundary viral disease that affects birds, posing a serious threat to poultry production worldwide (1–10). The etiological cause of the disease is a pleomorphic enveloped virus, and the genome of NDV is a single-stranded, nonsegmented, negative-sense RNA virus. Most orders of birds can be infected by the virus, indicating that it has a broad host range (11–14). Additionally, depopulation of the flocks infected by NDV has the potential to reduce food supplies, which has a severe influence on both human welfare and economic prosperity (15–19). The World Organisation for Animal Health (Office International des Epizooties [OIE]) has placed NDV, also known as avian paramyxovirus type 1 (APMV-1), on List A in the old classification of diseases notifiable to the OIE (11, 20). Since NDV was first described in 1926, 9 genotypes of class I viruses and 10 genotypes of class II viruses have been detected, indicating a diversified group of viruses that have emerged and reemerged (21–28). According to the most recent updated system of the NDV classification nomenclature (29), class I is considered to include one genotype, but there are more within class II. Newcastle disease can be controlled using vaccines in combination with good management and biosecurity. Vaccine failure is, however, a persistent problem in several areas owing to antigenic differences between the utilized vaccine and field strains, despite the fact that they are of the same serotype (11, 30–35). Moreover, circumstantial evidence suggests that vaccinated chickens can serve as reservoirs for extremely virulent Newcastle disease viruses (vNDV) because vaccination has been shown to have no effect on the proliferation of these viruses in vaccinated flocks. Additionally, the presence of lentogenic NDV strains in chickens could be a risk factor for velogenic NDV outbreaks in the future (21, 36–39). Also, extensive vaccination against NDV resulted in increased immunological selection pressure from hosts, which aided NDV’s evolution (40). Furthermore, traditional vaccines are affordable and can be administered in a variety of ways, including by water or aerosol. On the other hand, the possibility of the live virus reverting to its virulent state is a risk that must be addressed (38). Another factor contributing to lower vaccine efficiency is the presence of antibodies—particularly maternally derived antibodies (MDAs)—in birds, which might neutralize the vaccine and hence impair its effectiveness (41, 42). The inactivated vaccine is time-consuming and costly to produce; additionally, when chickens are inoculated, their immune systems may develop an atypical immune response to ND (43) as a result of a weakness in cell-mediated immune responses (44) and/or an insufficient humoral immune response (45). Newcastle disease therefore frequently reemerges in many countries where extensive immunization has been applied (11, 12, 37, 41, 46), demonstrating that vaccination alone, without improvements in immunological efficacy, may not be adequate to manage ND.

Genotype VII is of special significance because it has been linked to a number of recent outbreaks in South America, the Middle East, Africa, and Asia (21, 26, 40, 47–51). After decades of research and development aimed at creating the best possible ND vaccine, new concepts and improved formulations for vaccine generation are still needed (17, 41, 52, 53). In several live-vector or inactivated NDV vaccines, our research team used biological adjuvants, such as chicken granulocyte monocyte colony-stimulating factor (ChGM-CSF), chicken melanoma differentiation-associated protein 5 (MDA5), and chicken interferon (IFN)-stimulated gene 12-2 (ISG 12 [2]), which have been proven to improve the vaccine’s immunological effectiveness in chickens (1, 53–55). ChGM-CSF is a potent cytokine that regulates the maturation of granulocytes and macrophages from hematopoietic progenitors as well as improving the mature immune system’s response to antigens (56). Furthermore, in a recent chicken vaccination trial in which ChGM-CSF was used as an adjuvant, the kinetics and magnitude of the antibody response improved (55–58).

The goal of this study was to use immunostimulant ChGM-CSF bio-adjuvant with HN epitope of the genotype VII C22-strain to enhance chickens’ immunological genetic resistance, therefore improving the immunological effectiveness of a transgenic live recombinant NDV genotype-matched transgenic vaccine to overcome the limitations imposed by cytokines’ short half-life. As there is no preexisting human adenoviral immunity found in animals, recombinant adenovirus type 5 is considered to be more immunogenic and stable. This avoids preexisting antibodies either to the MDAs or in vaccinated chickens and is proposed as a promising potent transgenic virulent field-match vaccine, while also guaranteeing that no antibiotic resistance gene remains with the live vector. Therefore, the evolutionary process of NDV can be slowed down or even stopped, resulting in improved poultry health, NDV eradication, and protected economic livelihoods around the world.

## RESULTS

### Construction of the plasmids and rescuing viruses.

**FIG 1:**
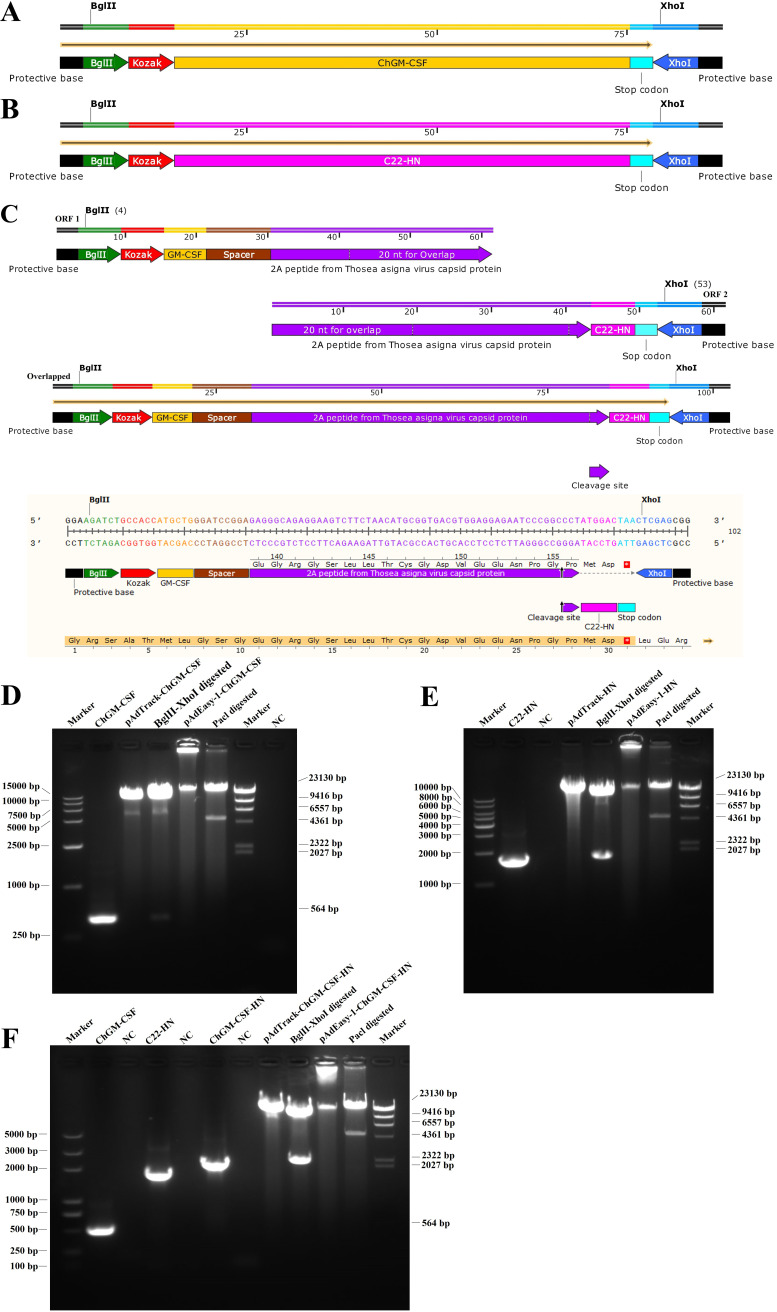
(A to C) Diagrams of the construction of (A) ChGM-CSF (435-bp), (B) C22-HN (1,716-bp), and (C) ChGM-CSF-HN (2,211-bp). (D to F) Results following construction of the genes (D) ChGM-CSF, (E) C22-HN, and (F) ChGM-CSF-HN in the pAdTrack-CMV backbone and subsequent generation of those genes in the pAdEasy-1 adenoviral backbone plasmid, with a comprehensive unique restriction enzyme digestion analysis carried out in order to determine whether constructs were successful. The T2A peptide from the *Thosea asigna* virus capsid protein enables the expression of two functional proteins through eukaryotic ribosomes that fail to insert a peptide bond between the glycine and proline residues, yielding separate polypeptides.

The diagrams of the construction of the ChGM-CSF, C22-HN, and ChGM-CSF-HN are shown in Fig. 1A to C, respectively. In addition, as shown in Fig. 1D to F, the successful insertion of the additional transcription units of ChGM-CSF, C22-HN, and ChGM-CSF-HN into pAdTrack-CMV and, afterward, into the adenoviral backbone plasmid pAdEasy-1 were examined using unique restriction enzymes and gel electrophoresis.

**FIG 2:**
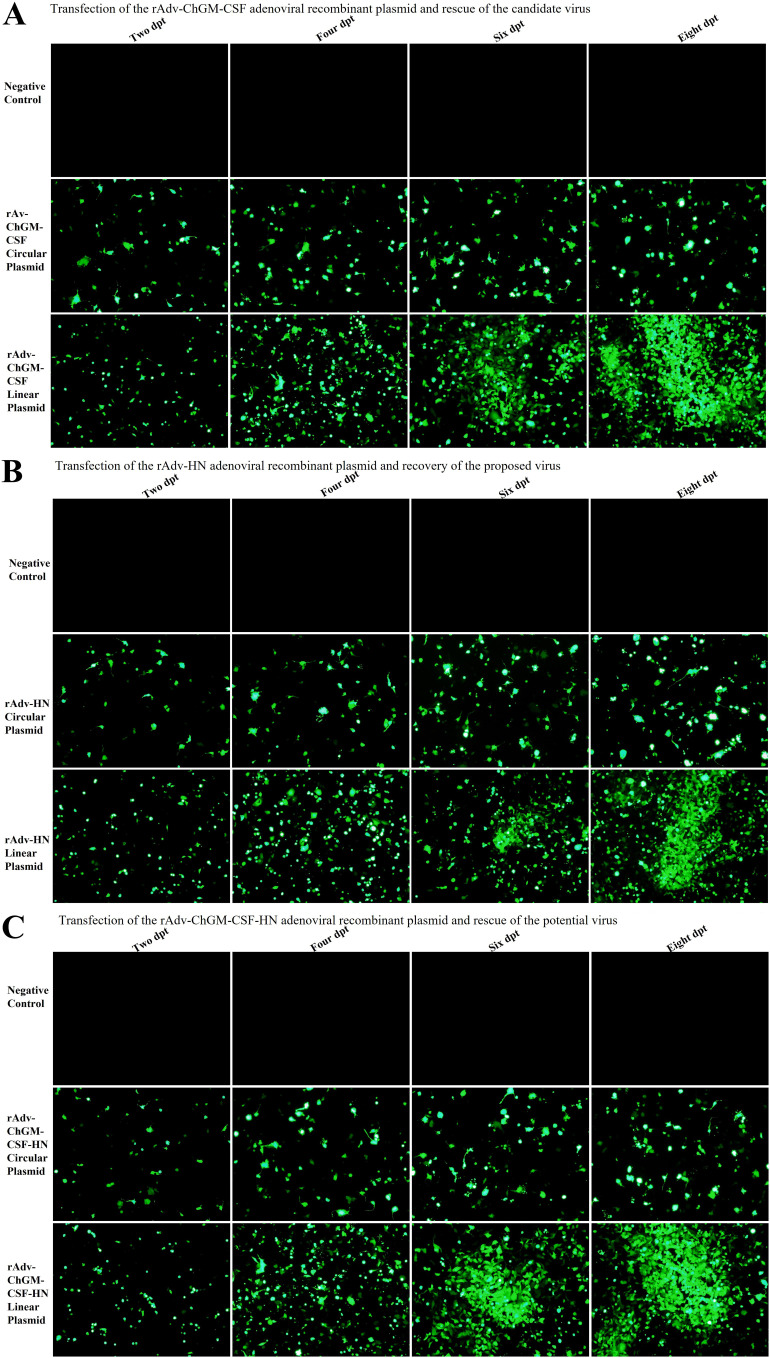
(A to C) HEK-293A cells at 2, 4, 6, and 8 days after being transfected with circularly or linearly designed plasmids incorporating (A) rAdv-ChGM-CSF, (B) rAdv-HN, and (C) rAdv-ChGM-CSF-HN. Between 6 and 8 days after being transfected by the plasmids that had undergone overnight PacI enzyme digestion, the majority of the viruses were successfully recovered, and comet-like adenovirus-producing foci started to become visible.

Furthermore, as shown in Fig. 2A to C, the PacI-linearized adenoviral backbone plasmids pAdEasy-1 that had been transfected into HEK-293A were recovered in an average of 6 to 8 days after the transfection. These rescued recombinant viruses were given the names rAdv-ChGM-CSF, rAdv-HN, and rAdv-ChGM-CSF-HN.

### Biological activities of the rescued viruses.

**FIG 3:**
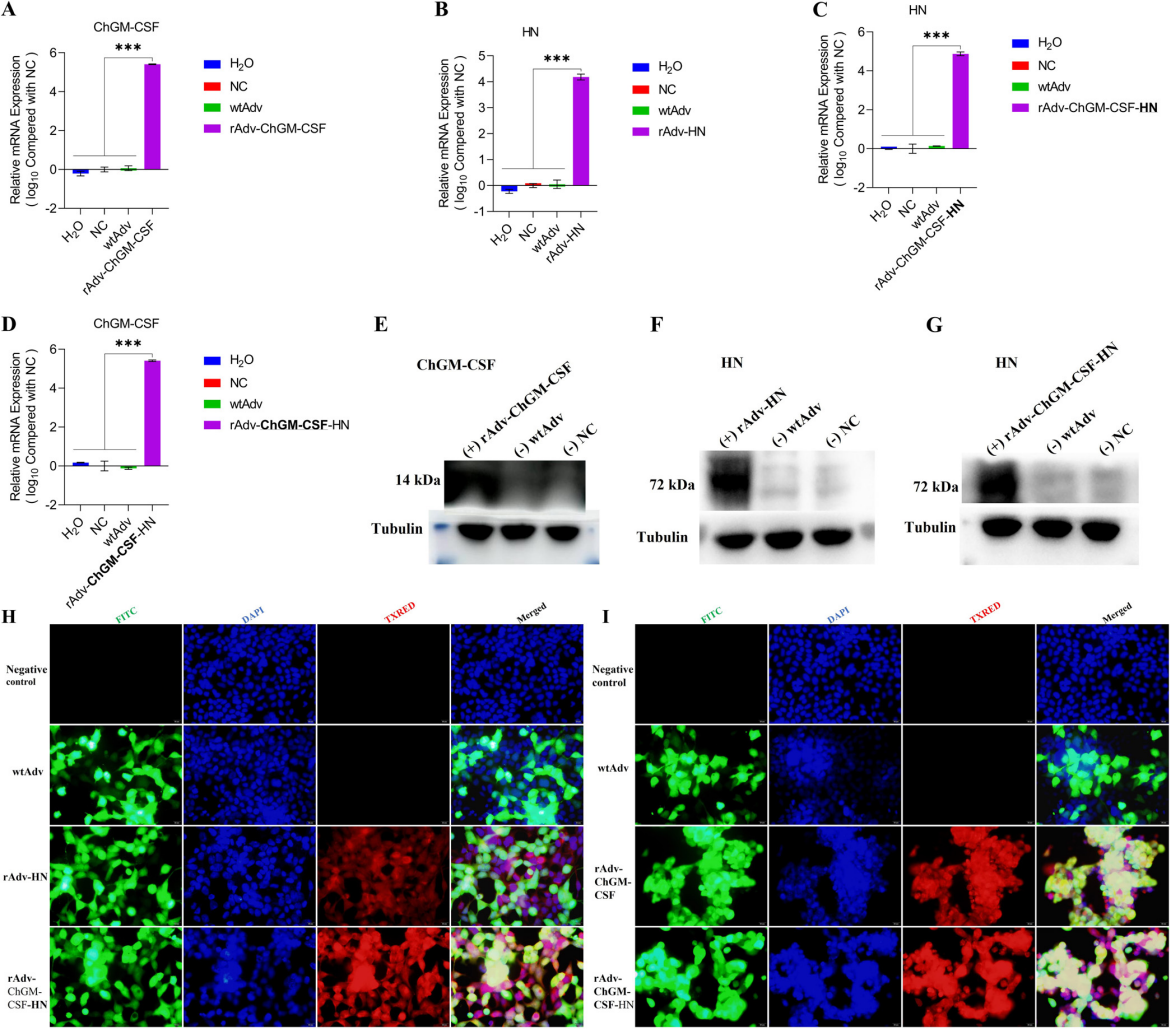
The HEK-293A cells were infected with an MOI of 0.01 of wtAdv, rAdv-ChGM-CSF, rAdv-HN, and rAdv-ChGM-CSF-HN. (A to D) After 24 h postinfection (hpi), the relative mRNA gene expression was assessed for the ChGM-CSF and the C22-HN. (E to G) Results of the Western blot for both ChGM-CSF and C22-HN. (H) Moreover, the C22-HN IFA was proved to exist by both the rAdv-HN and the rAdv-ChGM-CSF-HN. (I) rAdv-ChGM-CSF and rAdv-ChGM-CSF-HN, which are representative of ChGM-CSF IFA. Each bar indicates the mean ± one standard deviation. Statistics-based differences are marked by asterisks (***, *P < *0.001).

According to the findings, which are depicted in Fig. 3A to D, the candidate recovered viruses, namely, rAdv-ChGM-CSF, rAdv-HN, and rAdv-ChGM-CSF-HN, had highly significant (*P < *0.001) mRNA gene expression differences in the HEK-293A-infected cells compared to the wtAdv-infected cells and nonchallenged [NC] noninfected cells. In addition, in the HEK-293A-infected cells, the results shown in Fig. 3E to G reveal that the novel rescued viruses successfully produce the protein that is encoded by the new transcriptional units that have been inserted. Moreover, the detection of the immunofluorescent antibody (IFA) of rAdv-HN and rAdv-ChGM-CSF-HN was carried out in HEK-293A-infected cells at 24 h postinfection, as shown in Fig. 3H and I, which demonstrated the IFA of rAdv-ChGM-CSF and rAdv-ChGM-CSF-HN.

**FIG 4:**
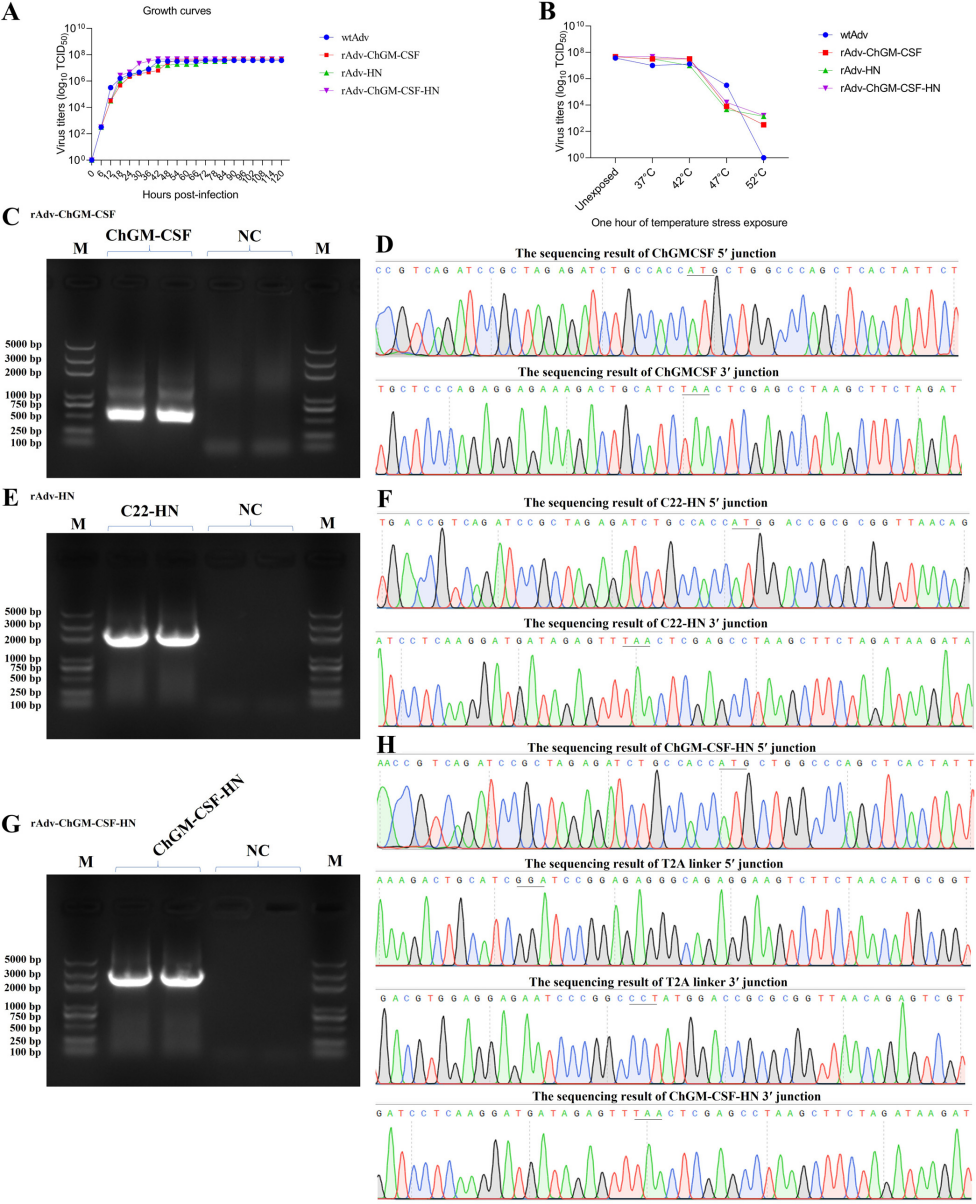
(A and B) HEK-293A cells infected with an MOI of 2 of wtAdv, rAdv-ChGM-CSF, rAdv-HN, and rAdv-ChGM-CSF-HN for up to 5 days in order to assess their growth curves (A), as well as their thermostability while they were exposed for an hour to various temperature points (B). In both the growth curves and the thermostability tests, the statistics among the treated groups did not show any significant differences. (C to H) After 20 rounds of replication, the genetic stability of the ChGM-CSF (435 bp) (C and D), C22-HN (1,716 bp) (E and F), and ChGM-CSF-HN (2,211 bp) (G and H) target genes was verified by using a PCR-based amplification process and their sequencing.

As shown in Fig. 4A, the growth curves of the newly generated viruses were evaluated in HEK-293A cells, and the results revealed that there was no statistically significant difference between the treatments. The newly created viruses, on the other hand, are thermostable when they are exposed to different temperature points, as shown in Fig. 4B, and the results were then found to be nonsignificant between them. In addition, the genes of interest (GOI) inserted into the novel viruses, named rAdv-ChGM-CSF, rAdv-HN, and rAdv-ChGM-CSF-HN, were considered to be genetically stable after 20 rounds of replication in the HEK-293A cells; this conclusion was reached on the basis of the PCR and its sequencing results, which are shown in Fig. 4C and D, E and F, and G and H, respectively.

**FIG 5:**
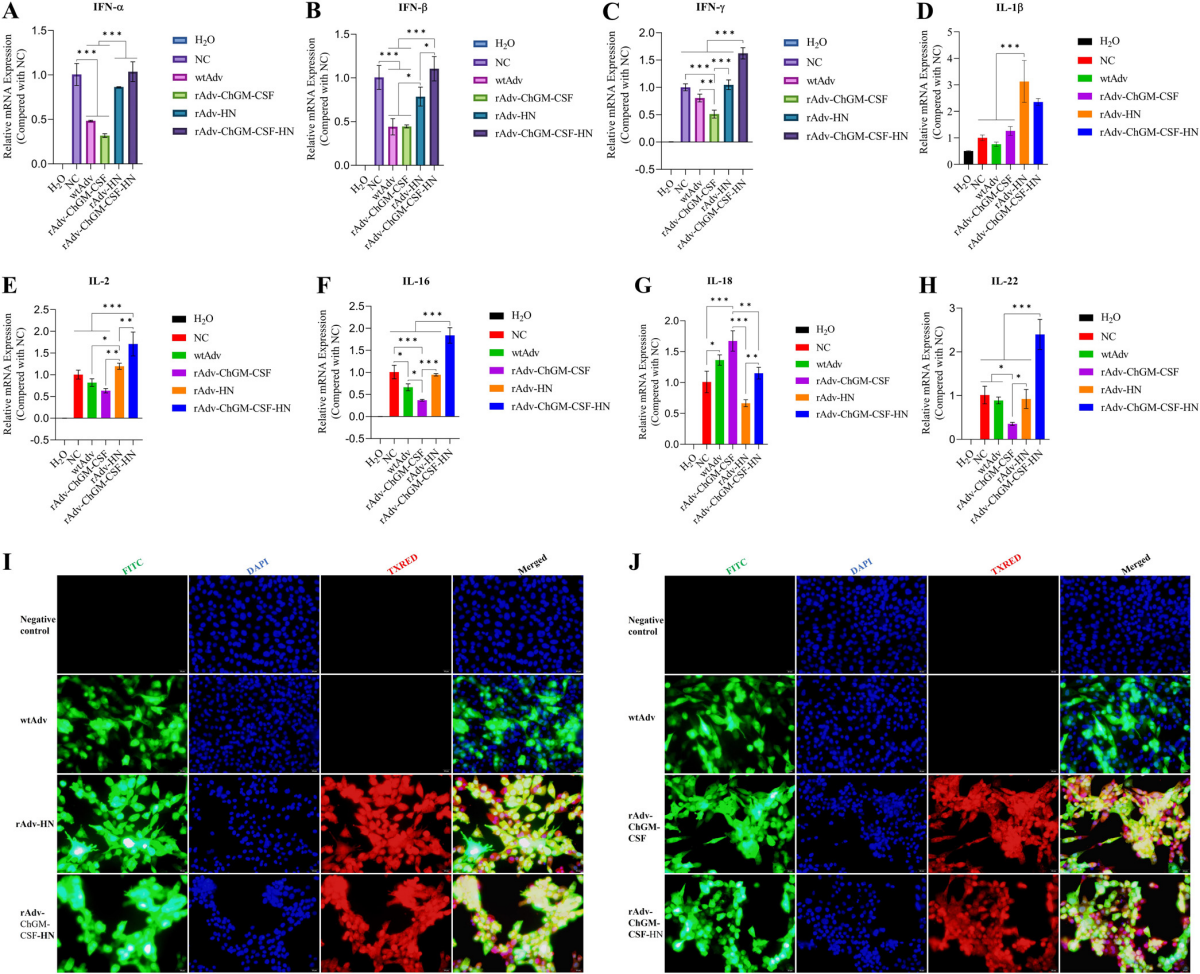
The DF1 cells were treated with an MOI of 2 of wtAdv, rAdv-ChGM-CSF, rAdv-HN, and rAdv-ChGM-CSF-HN. (A to H) After 48 hpi, the relative mRNA gene expression of (A) INF-α, (B) INF-β, and (C) INF-γ was evaluated in comparison to the negative control, as well as the (D) IL-1β, (E) IL-2, (F) IL-16, (G) IL-18, and (H) IL-22. (I and J) On the other hand, in DF1 cells that were inoculated in the same manner and at the same time point as shown above, the IFA of the C22-HN was detected in cells treated with rAdv-HN and rAdv-ChGM-CSF-HN (I), whereas the IFA of ChGM-CSF was observed in cells treated with rAdv-ChGM-CSF and rAdv-ChGM-CSF-HN (J).

As shown in Fig. 5A, the results of this study revealed that INF-α was significantly greater (*P < *0.001) in the NC chicken embryo fibroblast cells (DF1) cells or rAdv-HN- and rAdv-ChGM-CSF-HN-treated cells than in the wtAdv- and rAdv-ChGM-CSF DF1-treated cells. While the rAdv-HN-treated cells exhibit a significant difference (*P < *0.05) from the wtAdv- and rAdv-ChGM-CSF-treated cells, the INF-β levels in the NC DF1 cells and rAdv-ChGM-CSF-HN cells were significantly higher (*P < *0.001) than in the wtAdv- and rAdv-ChGM-CSF-treated cells. In addition, the cells that were treated with rAdv-ChGM-CSF-HN displayed a statistically significant difference (*P < *0.05) compared to the cells that were treated with rAdv-HN (Fig. 5B). Also, in the evaluation of the expression of INF-γ, the results revealed that the NC and rAdv-HN cells were determined to be extremely significantly different (*P < *0.001) from the wtAdv- and rAdv-ChGM-CSF-treated cells (Fig. 5C). The wtAdv-treated cells were found to be significantly higher (*P < *0.01) than the rAdv-ChGM-CSF-infected cells, while the rAdv-ChGM-CSF-HN-infected cells were found to be significantly different (*P < *0.001) than all examined DF1 cells, indicating that the candidate virus, namely, rAdv-ChGM-CSF-HN, had the ability to improve the immune response.

As shown in Fig. 5D, the levels of IL-1β were measured, and the results showed that the rAdv-HN-infected cells had considerably greater levels (*P < *0.001) than the NC cells and the wtAdv- and rAdv-ChGM-CSF-treated cells. On the other hand, cells treated with rAdv-ChGM-CSF-HN showed significant differences (*P < *0.01, *P < *0.001, and *P < *0.05) from the NC cells or those that were treated with wtAdv and rAdv-ChGM-CSF, respectively. The relative gene expressions of IL-2 were analyzed, and the results showed that the levels of IL-2 in the rAdv-HN-infected cells were significantly higher (*P < *0.05 and *P < *0.01) than those in the wtAdv- and rAdv-ChGM-CSF-treated cells, respectively. In addition, the superior stimulation of the cell’s immunology was detected when the cells treated with rAdv-ChGM-CSF-HN were found to be very different (*P < *0.01) from the rAdv-HN infected cells, and there were greater differences (*P < *0.001) compared to the NC cells and wtAdv- and rAdv-ChGM-CSF-treated cells (Fig. 5E). In the evaluation of IL-16, the findings showed that the NC and the rAdv-HN-treated cells had considerably greater levels (*P < *0.001) than the rAdv-ChGM-CSF-treated cells (Fig. 5F). On the other hand, the NC and wtAdv significantly (*P < *0.05) differed from the rAdv-ChGM-CSF-treated cells. While the cells treated with rAdv-ChGM-CSF-HN revealed a highly significant difference (*P < *0.001) from the cells receiving any of the other treatments, the results of this study demonstrated the effectiveness of rAdv-ChGM-CSF-HN in improving the immune system *in vitro*.

When the levels of IL-18 were studied, the rAdv-ChGM-CSF-infected cells exhibited highly significant amounts (*P < *0.001) compared to the NC and rAdv-HN-treated cells and (*P < *0.01) from the rAdv-ChGM-CSF-HN-treated cells. While the wtAdv significantly (*P < *0.05) differed from the NC, the rAdv-ChGM-CSF-HN-infected cells had a significantly higher among (*P < *0.01) than the rAdv-HN-infected cells (Fig. 5G). Furthermore, by assessing the IL-22, the findings demonstrated that the NC, wtAdv, and rAdv-HN had significant differences (*P < *0.05) from the rAdv-ChGM-CSF (Fig. 5H). The cells infected with the rAdv-ChGM-CSF-HN explained the highly significant difference (*P < *0.001) from all other treated cells, once again indicating the potential for improving the immune response *in vitro*. Additionally, DF1-infected cells were used to detect the IFA of rAdv-HN and rAdv-ChGM-CSF-HN at 48 h after infection, as shown in Fig. 5I, and Fig. 5J shows the IFA of rAdv-ChGM-CSF- and rAdv-ChGM-CSF-HN-infected cells.

### Serological testing.

**FIG 6:**
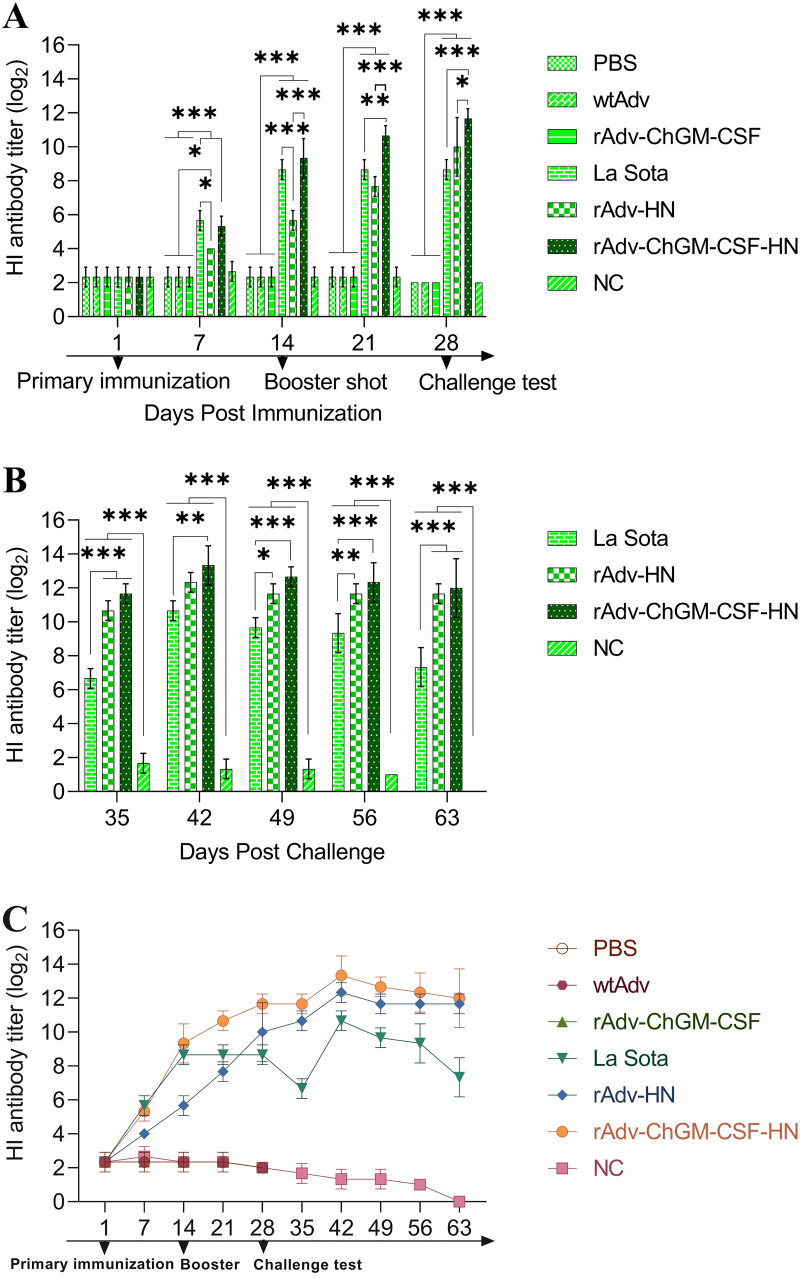
(A) The hemagglutination inhibition (HI) titers (log_2_) of the serum antibodies were utilized to determine the amount of the specific NDV antibodies present on day 1 before vaccination and at 7, 14, 21, and 28 days post-primary immunization. (B) The HI titers (log_2_) after the challenge test at 35, 42, 49, 56, and 63. (C) Whole HI titers (log_2_), i.e., before and after the challenge. Each bar indicates the mean ± one standard deviation. Statistics-based differences are marked by asterisks (*, *P < *0.05; **, *P < *0.01; ***, *P < *0.001).

As shown in Fig. 6A, prior to primary immunization, all chickens had low levels of NDV-specific MDAs, with a hemagglutination inhibition (HI) log_2_ mean value of 2.33 and were therefore considered negative for NDV-specific antibodies. With the exception of those in the control groups (nonchallenged [NC], control challenge [CC], wtAdv, and rAdv-ChGM-CSF), specific antibodies against NDV increased after 7 days post-primary immunization, particularly in the La Sota strain group, which had a significant HI log_2_ value mean of 5.67 (*P < *0.05) compared with the rAdv-HN group, which had a HI log_2_ value mean of 4. On the other hand, there were no clinical or pathological abnormalities identified in chickens that were dissected 4 days after immunization. This suggests that all of the vaccines that were utilized are safe. However, the La Sota and rAdv-ChGM-CSF-HN groups, as well as the rAdv-HN group, were found to have significantly higher antibody titers (*P < *0.001 and *P < *0.05, respectively) than the unvaccinated groups. Moreover, 2 weeks post-primary immunization, the La Sota and rAdv-ChGM-CSF-HN groups exhibited significantly higher antibody titers (*P < *0.001) than the rAdv-HN group. At 1 week post-booster immunization, the rAdv-ChGM-CSF-HN group had an HI log_2_ value mean of 10.67 (*P < *0.001 and *P < *0.01, respectively), which was extremely significant compared to the rAdv-HN group mean of 7.67 and the La Sota group mean of 8.67. On day 28 post-primary immunization, the rAdv-ChGM-CSF-HN group had significantly higher antibody titers, with an HI log_2_ value mean of 11.67 (*P < *0.05 and *P < *0.001, respectively) compared to the rAdv-HN group (HI log_2_ mean of 10 and La Sota log_2_ mean of 8.67). In contrast, the La Sota group antibody titers remained stable at an HI log_2_ value mean of 8.67 at 21 and 28 days postvaccination (Fig. 6A). Additionally, as shown in Fig. 6B, at 7 dpc, the rAdv-HN and rAdv-ChGM-CSF-HN groups had log_2_ value means of 10.67 and 11.67, respectively, with a highly significant difference (*P < *0.001) from the La Sota group, which had a lower HI log_2_ value mean of 6.67. At 14 dpc, the rAdv-ChGM-CSF-HN group had a significantly higher HI log_2_ value mean of 13.33 (*P < *0.01) than the La Sota group’s log_2_ value mean of 10.67. Moreover, at 21 dpc, the rAdv-ChGM-CSF-HN and rAdv-HN groups had significantly higher HI log_2_ value means of 12.67 (*P < *0.001) and 11.67 (*P < *0.05), respectively, than the La Sota group log_2_ value of 9.67. As of 28 dpc, the rAdv-ChGM-CSF-HN and rAdv-HN groups had significantly higher HI log_2_ value means of 12.33 (*P < *0.001) and 11.67 (*P < *0.01), respectively, than the La Sota HI log_2_ value mean of 9.33. Furthermore, at 35 dpc, the rAdv-ChGM-CSF-HN and rAdv-HN groups had HI log_2_ means of 12 and 11.67, respectively, which were highly significant differences (*P < *0.001) compared to the La Sota HI log_2_ mean of 7.33 (Fig. 6B). As expected, anti-NDV humoral responses were unaffected by wild-type or rAdv-ChGM-CSF adenoviruses, with lower HI titers in the control groups. Both before and after challenge, the rAdv-ChGM-CSF-HN vaccine elicited significant levels and persistent antibody titers against NDV, indicating the immunomodulatory potential of the improved NDV vaccine compared to the La Sota and rAdv-HN vaccines (Fig. 6C).

### Cellular immunity.

**FIG 7:**
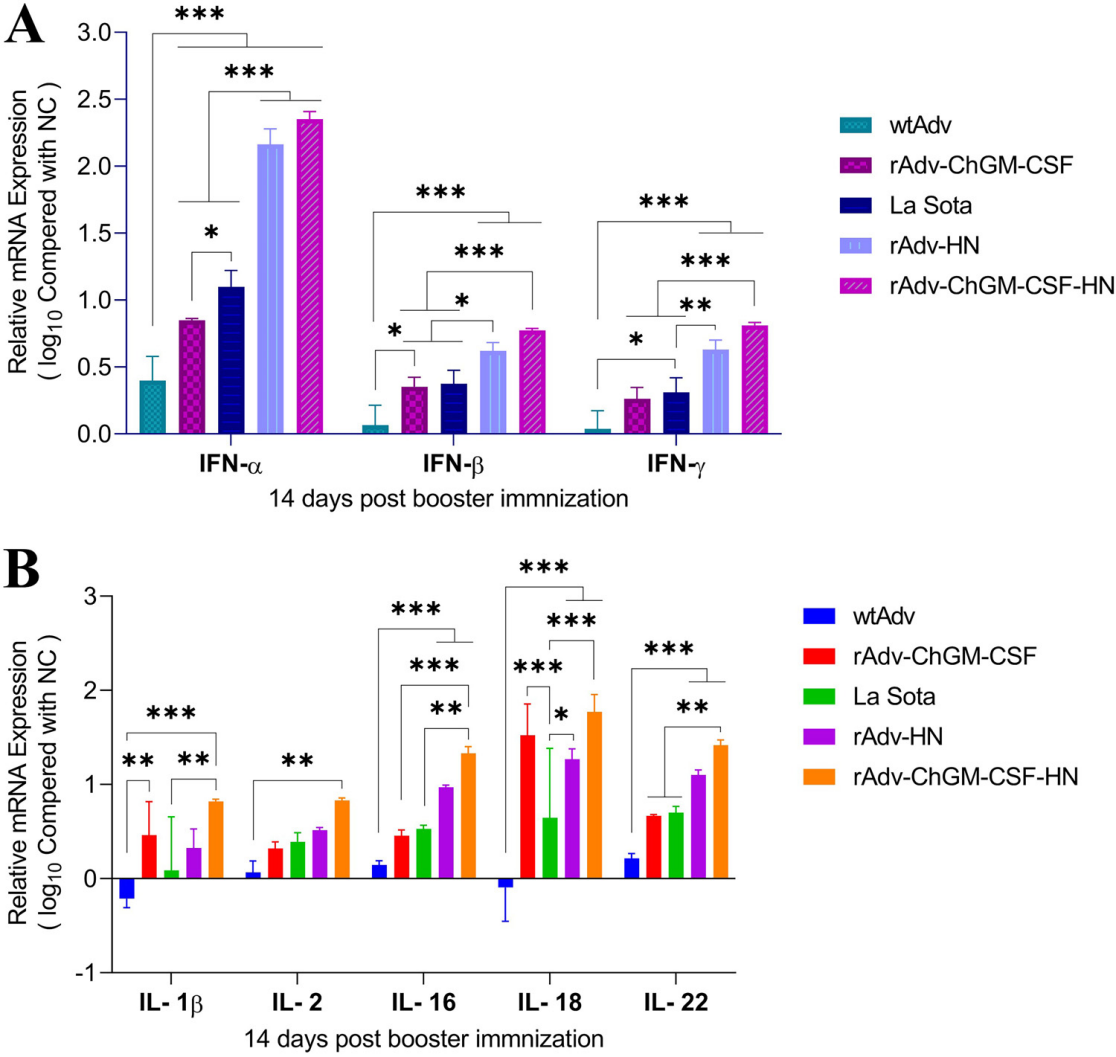
(A and B) At 14 days post-booster immunization, the 2^–ΔΔ^*^CT^* procedure was used in order to assess and estimate the fold expression of the genes in splenocytes that code for IFN-α, IFN-β, and IFN-γ A as well as IL-1β, IL-2, IL-16, IL-18, and IL-22 (B) in comparison with the NC group, with 28s serving as the reference gene. Each bar indicates the mean ± one standard deviation. Asterisks are used to indicate statistically significant differences between groups (*, *P < *0.05; **, *P < *0.01; ***, *P < *0.001).

As shown in Fig. 7A, the IFN-α levels in the groups treated with rAdv-ChGM-CSF, La Sota, rAdv-HN, and rAdv-ChGM-CSF-HN were significantly higher (*P < *0.001) than those of the wtAdv group. In contrast, groups treated with rAdv-HN and rAdv-ChGM-CSF-HN exhibited the highest significance value (*P < *0.001) compared to the groups treated with rAdv-ChGM-CSF and La Sota. However, the La Sota group showed a significant difference (*P < *0.05) from the rAdv-ChGM-CSF group. Additionally, the IFN-β levels in the rAdv-HN and rAdv-ChGM-CSF-HN groups were significantly higher (*P < *0.001) than those in the wtAdv group, but the rAdv-ChGM-CSF-HN group had considerably greater levels of significance (*P < *0.001) than the rAdv-ChGM-CSF and La Sota groups. Aside from that, the rAdv-HN group had statistical significance (*P < *0.05) compared with the rAdv-ChGM-CSF- and La Sota-treated groups. However, the rAdv-ChGM-CSF-treated group revealed a significant value (*P < *0.05) compared to the wtAdv groups, and the rAdv-ChGM-CSF and La Sota groups did not differ significantly. Furthermore, IFN-γ levels were significantly greater (*P < *0.001) in the rAdv-HN and rAdv-ChGM-CSF-HN groups than in the wtAdv group. In contrast, the rAdv-ChGM-CSF-HN group demonstrated substantial statistical significance (*P < *0.001) in comparison to the rAdv-ChGM-CSF and La Sota groups. Additionally, the rAdv-HN group demonstrated statistical significance (*P < *0.01) compared to the La Sota group. However, the La Sota group showed a significant difference (*P < *0.05) from the wtAdv group. Also, no statistically significant difference existed between the rAdv-ChGM-CSF and La Sota groups (Fig. 7A).

As shown in Fig. 7B, the rAdv-ChGM-CSF-HN group seemed to have the highest levels of IL-1β, showing statistically significant differences (*P < *0.001) from the wtAdv group. On the other hand, the rAdv-ChGM-CSF-HN group had significantly higher (*P < *0.01) levels compared to the La Sota group. It was shown that the rAdv-ChGM-CSF group had significantly higher levels of IL-1β (*P < *0.01) than the wtAdv group. Moreover, there was no statistically significant difference between the La Sota and rAdv-HN groups, or between the wtAdv and rAdv-ChGM-CSF groups. Also, compared to rAdv-ChGM-CSF and rAdv-HN, rAdv-ChGM-CSF-HN showed no statistically significant difference. Furthermore, the rAdv-ChGM-CSF-HN group showed an exclusive rise in IL-2 levels at the *P < *0.01 level of significance compared to the wtAdv group, while all other findings were nonsignificant (Fig. 7B). Also, the highest levels of IL-16 were seen in the rAdv-HN and rAdv-ChGM-CSF-HN groups, with a significant difference of (*P < *0.001) compared to the wtAdv group. Additionally, the rAdv-ChGM-CSF-HN group revealed significant differences (*P < *0.001) compared to the rAdv-ChGM-CSF group, while the significance value was (*P < *0.01) compared to the La Sota group. When comparing the rAdv-ChGM-CSF and La Sota groups to each other and to the wtAdv group, there were no statistically significant differences (Fig. 7B). Compared to the control group (wtAdv), those treated with rAdv-ChGM-CSF, rAdv-HN, and rAdv-ChGM-CSF-HN had significantly (*P < *0.001) higher levels of IL-18, as opposed to the statistically significant difference (*P < *0.01) from the La Sota-treated group, while the IL-18 levels in the rAdv-ChGM-CSF and rAdv-ChGM-CSF-HN groups were higher than and statistically significant (*P < *0.001) compared to the La Sota group. On the other hand, the rAdv-HN group shows significant differences (*P < *0.05) compared to the La Sota group (Fig. 7B). The IL-22 levels were found to be higher in the rAdv-HN and rAdv-ChGM-CSF-HN groups, with (*P < *0.001) statistically significant differences compared to the wtAdv group. However, the rAdv-ChGM-CSF-HN group had higher levels of IL-22 with significant differences (*P < *0.01) compared to the rAdv-ChGM-CSF and La Sota groups. Also, there were no significant differences between the rAdv-ChGM-CSF and La Sota groups compared to each other and to the wtAdv group (Fig. 7B). In summary, without specific antigen stimulation, ChGM-CSF has a minor influence on immune responses. Comparatively, combining ChGM-CSF with the HN epitope of the NDV genotype VII-C22 strain (rAdv-ChGM-CSF-HN) improved its potential capability to stimulate the immune system more than when used alone.

### Protective effectiveness toward vNDV challenge.

**FIG 8:**
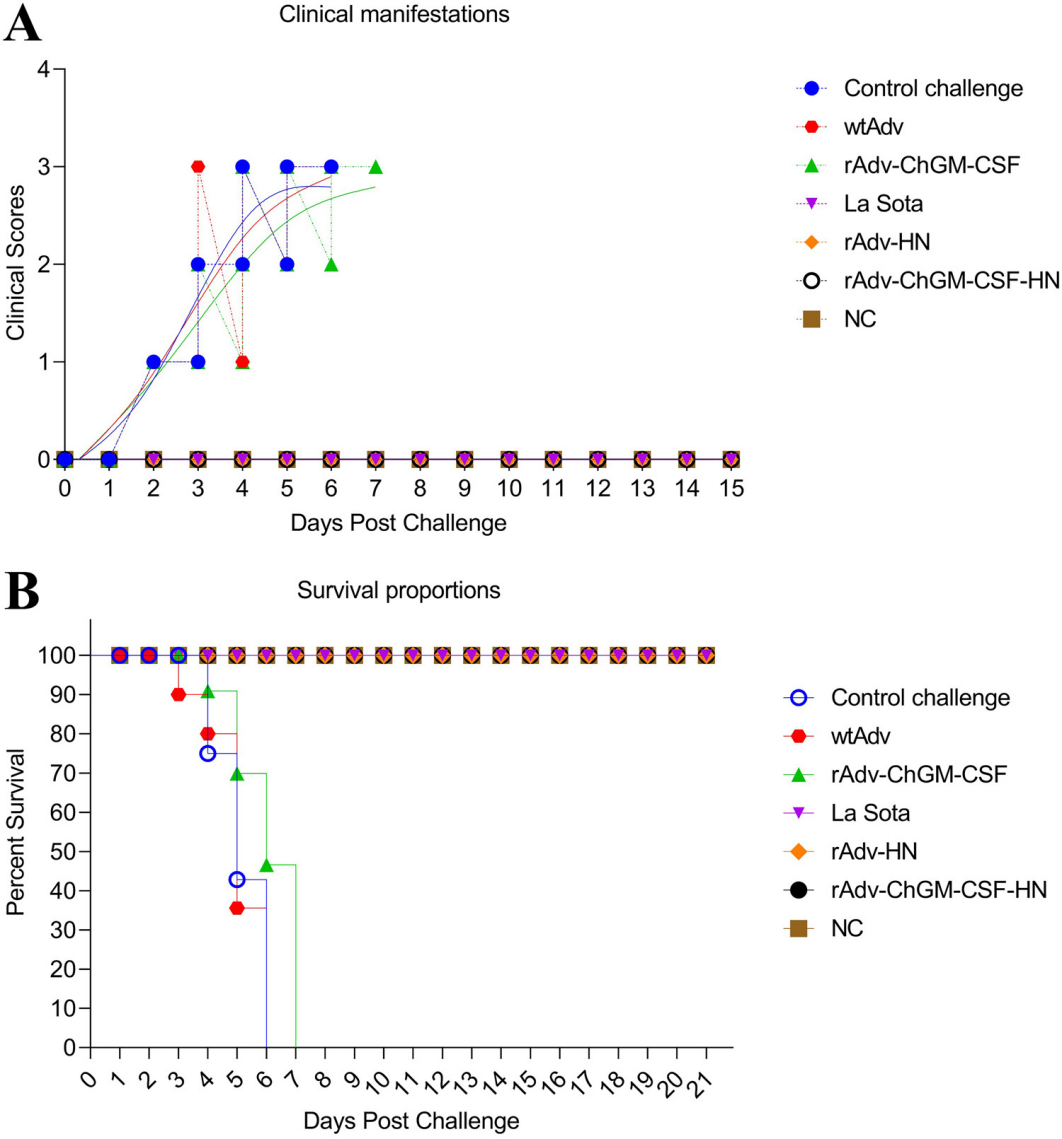
(A) Clinical manifestation scores of the chickens postchallenge. (B) The survival rates are used to demonstrate the effectiveness of the immunization in terms of safeguarding against disease.

Until the experiment was completed, there were no clinical signs of disease or mortality among the vaccinated chickens in the La Sota, rAdv-HN, and rAdv-ChGM-CSF-HN groups or in the NC group. Meanwhile, unvaccinated chickens in the control challenge (CC), wtAdv, and rAdv-ChGM-CSF groups were completely susceptible to the challenge, exhibiting daily clinical manifestations such as depression and conjunctivitis at 2 dpc, followed by acute ND clinical signs, including anorexia, distress ruffled feathers, severe conjunctivitis, dehydration and prostration, greenish diarrhea, trembling, and death from 3 to 7 dpc, as shown in Fig. 8A. The unvaccinated groups had mortality rates as early as 3 dpc, and the wtAdv group had three deaths. Moreover, four chickens died in the CC group 4 dpc, while two died in the wtAdv or rAdv-ChGM-CSF groups. Additionally, three chickens died in the wtAdv and rAdv-ChGM-CSF groups at 5 dpc, but five died in the CC group. Nonetheless, all chickens died in the CC and wtAdv groups at 6 dpc, whereas none died in the rAdv-ChGM-CSF group, and all the remaining chickens in the rAdv-ChGM-CSF group died at 7 dpc (Fig. 8B).

**FIG 9:**
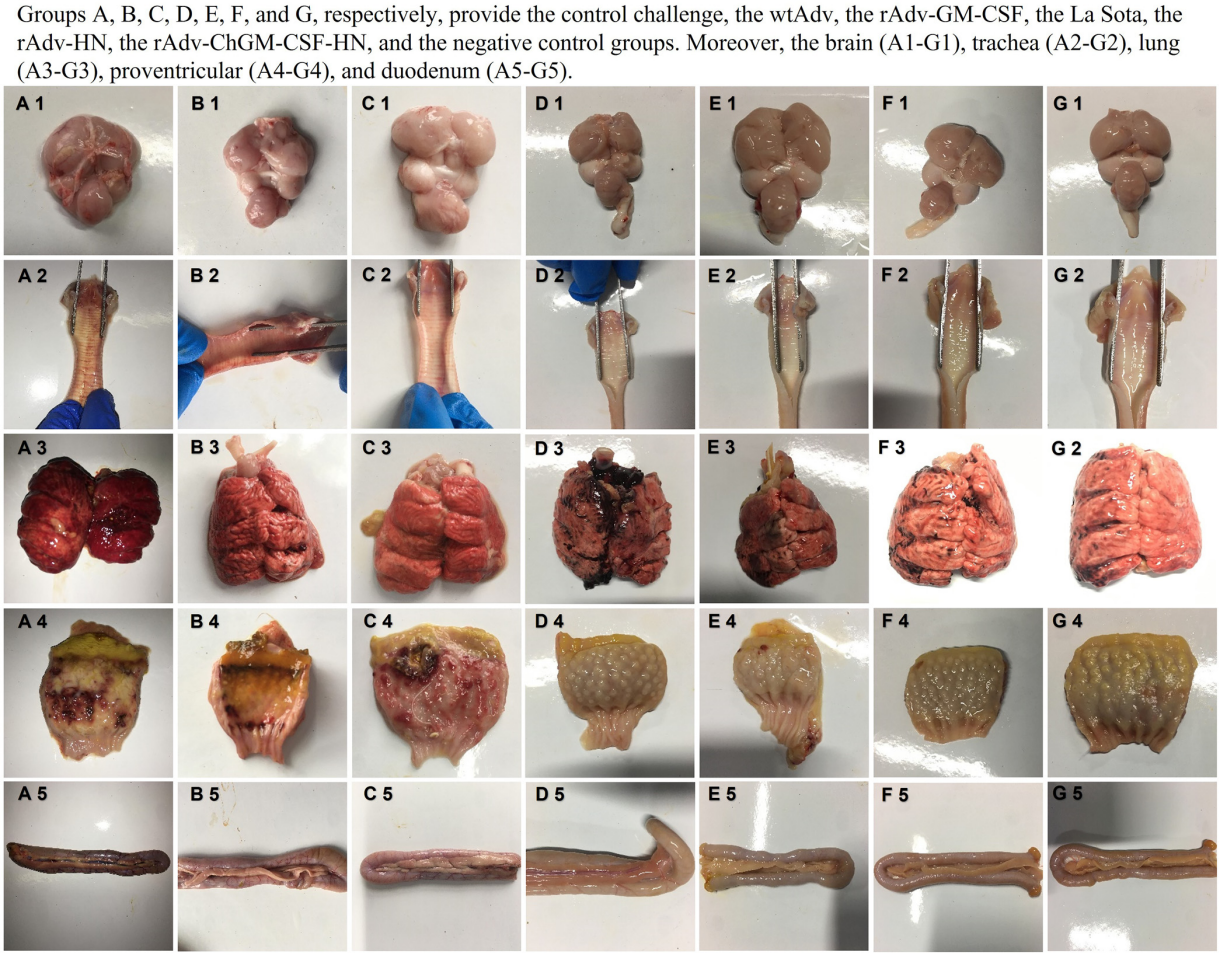
(A to C) Gross lesions of the control challenge (A), wtAdv (B), and rAdv-GM-CSF (C) groups; congested blood vessels, hemorrhages, and/or tissue damage were observed on the brain, lung, serosal surfaces of the trachea, glandular stomach papilla, and duodenum. (D) Only gross lung lesions are shown in the La Sota group. (E to G) The rAdv-HN group (E) had both gross lung lesions and focal hemorrhages in the glandular stomach papilla, whereas the rAdv-ChGM-CSF-HN group (F) had no gross lesions compared with the negative control group (G) showing normal organs.

As shown in Fig. 9, during necropsy at 5 dpc in the unvaccinated control groups, congested blood vessels, hemorrhages, and/or tissue damage were observed on the brain, lung, serosal surfaces of the trachea, glandular stomach papilla, and duodenum, with the severity of such hemorrhages increasing and worsening as the illness progressed. On the other hand, the vaccinated groups, particularly the La Sota group, had only gross lung lesions, whereas the rAdv-HN group had both gross lung lesions and focal hemorrhages in the proventriculus, while compared to the NC group with normal organs, the rAdv-ChGM-CSF-HN group had no gross lesions, indicating that the rAdv-ChGM-CSF-HN vaccine, which contains expressed ChGM-CSF, resulted in many fewer gross lesions in chickens and that the mitigation was even stronger (Fig. 9).

**FIG 10:**
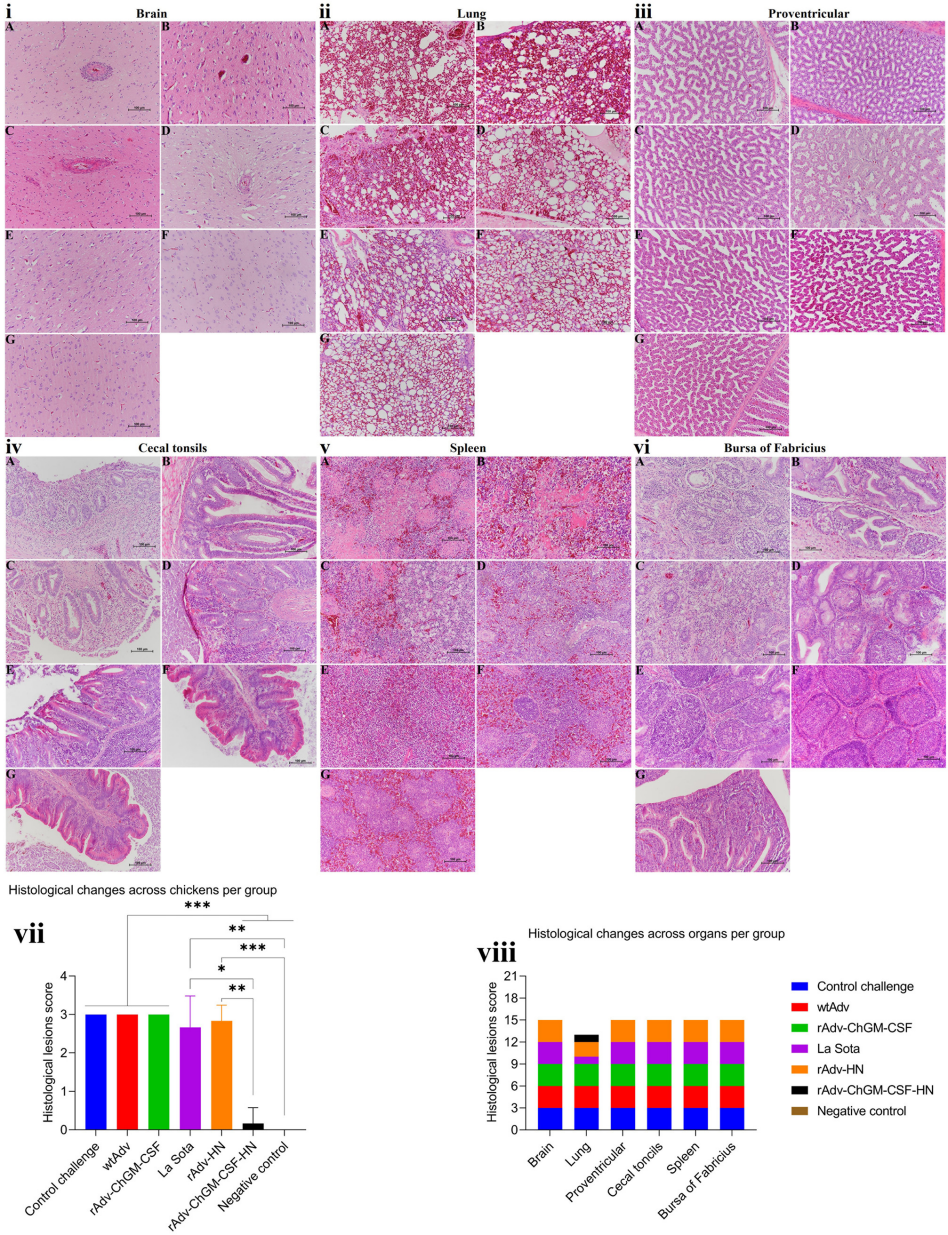
(i) (A to C) Histopathology examination of the brains in the control challenge (A), wtAdv (B), and rAdv-GM-CSF (C) groups showed lymphocytic perivascular cuffing, congestion, gliosis, and spongiosis associated with neuronal necrosis. (D) Congested blood vessels with mild perivascular edema and spongiosis associated with neuronal necrosis in the La Sota group brain. (E) The rAdv-HN group brain shows congested blood vessels with mild perivascular edema and neuronal necrosis. (F and G) The rAdv-GM-CSF-HN group brain (F)and the negative control group brain (G) both appear to have normal structures. (ii) (A and B) Histopathology examination of the control challenge and wtAdv group lungs shows congested blood vessels, edema, and necrotizing pneumonia associated with interstitial inflammatory cell infiltration and narrowing of the alveolar space. (C) In the rAdv-GM-CSF group, congested blood vessels, interstitial hyperplasia, and necrotizing pneumonia are associated with interstitial inflammatory cell infiltration and alveolar space narrowing. (D) La Sota group lung, showing mildly congested blood vessels and edema. (E) A lung from the rAdv-HN group with mildly congested blood vessels, hemorrhage, and interstitial inflammatory cell infiltration. (F) Lung from the rAdv-GM-CSF-HN group with mildly congested blood vessels and normal alveolar structures. (G) The lung of the negative-control group has congested blood vessels with alveolar emphysema. (iii) (A) In the control challenge group, proventricular histopathology shows mildly congested blood vessels and severe necrotic foci. (B and C) Proventricular tissue from the (B) wtAdv and (C) rAdv-GM-CSF groups shows mildly congested blood vessels and moderate lymphocytic inflammatory reactions associated with severe necrotic foci. (D) The proventricular glandular epithelium has been replaced by hyperplastic ductal epithelium (ductal epithelial metaplasia). (E) rAdv-HN group proventricular structures appear normal with mild necrotic foci. (F and G) In the rAdv-GM-CSF-HN group(F) and in the negative control group (G), proventricular structures appear normal. (iv) (A to C) Histopathology examination of the cecal tonsils of the control challenge (A), wtAdv (B), and rAdv-GM-CSF (C) groups shows lymphocyte infiltration and degeneration associated with severe necrotic enteritis. (D) La Sota group cecal tonsils show moderate lymphocyte proliferation associated with severe necrotizing enteritis. (E) The rAdv-HN group cecal tonsils showed severe lymphocyte proliferation and epithelial necrosis. (F and G) rAdv-GM-CSF-HN cecal tonsils (F) and negative control group cecal tonsils (G) both show apparently normal structures. (v) (A to C) Histopathology examination of the spleens of the control challenge (A), wtAdv (B), and rAdv-GM-CSF (C) groups shows severe congested blood vessels, hemorrhage, lymphocyte depletion, edema, and severe necrosis associated with undistinguishable white and red pulp compartments. (D) La Sota group spleen showing severe congested blood vessels, hemorrhage, lymphocyte depletion, central artery, periarteriolar lymphatic sheath, and necrosis associated with undistinguishable white and red pulp. (E) Spleen from the rAdv-HN group with severe lymphocyte proliferation, hemorrhage, and a periarteriolar lymphatic sheath associated with indistinguishable pulp compartments. (F) rAdv-GM-CSF-HN group spleen with distinct white and red pulp compartments, a lymph nodule, a central artery, a periarteriolar lymphatic sheath, and a periellipsoidal lymphatic sheath. (G) Normal negative-control group spleen with distinct white and red pulp compartments, a central artery, and a periellipsoidal lymphatic sheath. (vi) (A to C) Histopathology examination of control challenge (A), wtAdv (B), and rAdv-GM-CSF (C) groups bursa of Fabricius shows severe lymphocyte depletion, degeneration with interfollicular congestion, and necrosis. (D) La Sota group bursa of Fabricius showing proliferation of follicular cells with intrafollicular necrosis. (E) The rAdv-HN group bursa of Fabricius shows proliferation of follicular cells with mild intrafollicular necrosis. (F and G) The rAdv-GM-CSF-HN bursa of Fabricius (F) and the negative-control group bursa of Fabricius (G), which show apparently normal structures. (vii) There is a significant and consistent difference in the histological change scores of the tissues among chickens per group. (viii) Histological change scores across organs in each group. The statistically significant differences are shown as *, *P < *0.05; **, *P < *0.01; and ***, *P < *0.001.

As shown in Fig. 10i, the microscopic lesions in the unvaccinated groups, CC, wtAdv, and rAdv-ChGM-CSF, were brain had lymphocytic perivascular cuffing, gliosis, congested blood vessels (BVs), and spongiosis associated with neuronal necrosis; lung had congested BVs, edema, interstitial hyperplasia, and necrotizing pneumonia associated with interstitial inflammatory cell infiltration and narrowing of alveolar space (Fig. 10ii); congested BVs in the proventriculus and moderate lymphocytic inflammatory reaction associated with sever necrotic foci (Fig. 10iii); cecal tonsils had lymphocyte depletion, degeneration, and sever necrotic enteritis (Fig. 10iv); severely congested spleen BVs, hemorrhage, edema, lymphocyte depletion, and severe necrosis associated with undistinguishable white and red pulps compartments (Fig. 10v); and bursa showing severe lymphocyte depletion, interfollicular congestion with follicular degeneration, and necrosis (Fig. 10vi). As displayed in Fig. 10i, in the vaccinated groups, namely, the La Sota group, the brain had congested BVs, mild perivascular edema, and spongiosis associated with neuronal necrosis; the lung had congested BVs and edema (Fig. 10ii); the proventriculus showed replacement of glandular epithelium by hyperplastic ductal epithelium (ductal epithelial metaplasia) (Fig. 10iii); the cecal tonsils had moderate lymphocyte proliferation associated with severe necrotic enteritis (Fig. 10iv); the spleen had severe congested BVs, hemorrhage, lymphocyte depletion, central artery, periarteriolar lymphatic sheath, and necrosis associated with undistinguishable white and red pulps (Fig. 10v); and the bursa had proliferation of the follicular cells with intrafollicular necrosis (Fig. 10vi). Also, in the rAdv-HN group, the brain had apparently normal structures, congested BVs with mild perivascular edema, and neuronal necrosis (Fig. 10i); the lung had mildly congested BVs, hemorrhage, and interstitial inflammatory cell infiltration (Fig. 10ii); the proventriculus had apparently normal structures with mild necrotic foci (Fig. 10iii); the cecal tonsils showed severe lymphocyte proliferation and epithelial necrosis (Fig. 10iv); the spleen had severe lymphocyte proliferation, hemorrhage, central artery, and periarteriolar lymphatic sheath associated with undistinguishable pulp compartments (Fig. 10v); and the bursa had proliferation of follicular cells with mild intrafollicular necrosis (Fig. 10vi). Moreover, in the rAdv-ChGM-CSF-HN group, the brain, proventriculus, cecal tonsils, and bursa had apparently normal structures as shown in Fig. 10i, iii, iv, and vi, respectively, while the lung showed mildly congested BVs (Fig. 10ii); in the spleen, the white and red pulp compartments were clearly distinguished, as were the lymph nodule, central artery, periarteriolar lymphatic sheath, and periellipsoidal lymphatic sheath (Fig. 10v). The histology of the NC group revealed the brain, lung, proventriculus, cecal tonsil, spleen, and bursa to have apparently normal structures, as shown in Fig. 10i, ii, iii, iv, v, and vi, respectively. Besides the spleen, there were normal structures with white and red pulp compartments clearly distinguished, a central artery, and a periellipsoidal lymphatic sheath. In addition, the histological change scores across the tissues among chickens per group are shown in Fig. 10vii; the rAdv-ChGM-CSF-HN group and the NC group both demonstrate a highly significant and consistent difference in terms of low histological change scores (*P < *0.001) compared to the control challenge group, the wtAdv group, the rAdv-ChGM-CSF group, and the rAdv-HN group. Also, the rAdv-ChGM-CSF-HN group shows a statistically significant difference (*P < *0.05 and *P < *0.01, respectively) in comparison to the La Sota and rAdv-HN groups, whereas the NC group exhibits a statistically significant difference (*P < *0.01) compared to the La Sota group. Furthermore, the histological change scores across organs per group can be seen in Fig. 10viii.

### Tissues viral loads.

In order to measure the viral loads present in the various tissues, the quantitative PCR (qPCR) results were evaluated against the standard curve that had been created, which was *y* = 3.591 *x* = 41.544, with a correlation coefficient (*R*^2^) of 0.999 as shown in File S1 in the supplemental material.

**FIG 11:**
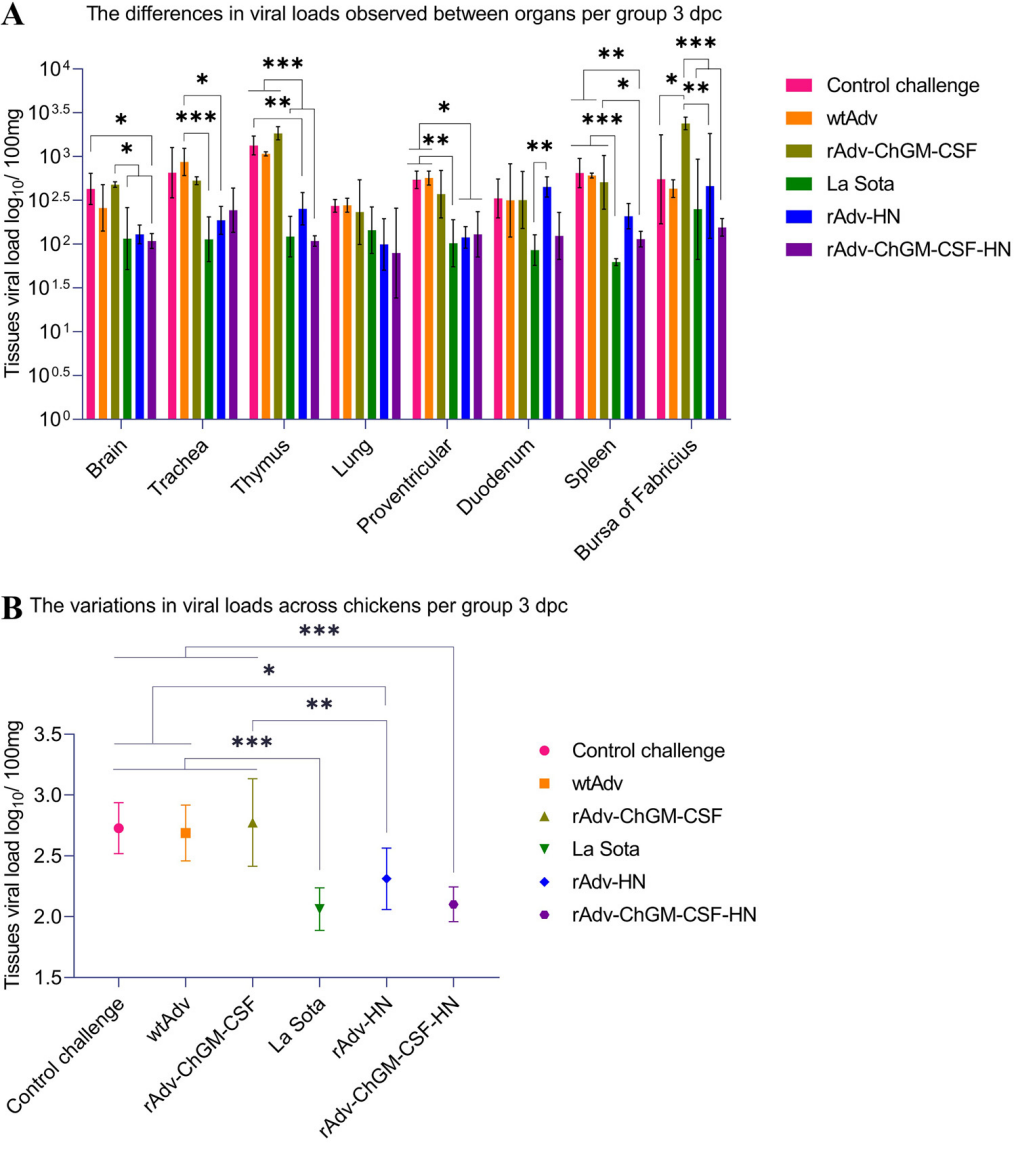
At 3 dpc, the viral loads in the various organs were quantified using SYBR green qPCR, which targeted the NP gene of the vNDV C22-strain with unique primers, and the results were evaluated against the standard curve, which had *y* = 3.591, *x* = 41.544, and a correlation coefficient (*R*^2^) of 0.999. (A) Differences in viral loads observed among various organs in the group. (B) Variations in viral loads across chickens per group. Each bar indicates the mean ± one standard deviation. The statistically significant differences are shown as *, *P < *0.05; **, *P < *0.01; and ***, *P < *0.001.

As shown in Fig. 11A, at 3 dpc, the brains of the La Sota and rAdv-ChGM-CSF-HN groups revealed significantly lower viral loads (*P < *0.05) than those of the rAdv-ChGM-CSF group, but the rAdv-ChGM-CSF-HN group revealed significantly lower viral loads (*P < *0.05) than the CC group, indicating that the rAdv-ChGM-CSF-HN group had the lowest viral loads in the brain across all the challenged treatments. The tracheal viral loads in the La Sota group were significantly lower (*P < *0.001) than those in the wtAdv group, and the tracheal viral loads in the rAdv-HN group were likewise significantly lower (*P < *0.05) (Fig. 11A). In the thymus, the La Sota and rAdv-ChGM-CSF-HN groups had significantly lower viral loads (*P < *0.001) than the CC, wtAdv, and rAdv-ChGM-CSF groups; however, the rAdv-HN group had significantly lower viral loads (*P < *0.01) than the CC group (Fig. 11A). In the lungs, there were no statistically significant differences between the challenge groups. In the proventriculus, the La Sota group exhibited significantly lower viral loads (*P < *0.01) than both the CC and wtAdv groups, whereas the rAdv-HN and rAdv-ChGM-CSF-HN groups had significantly lower viral loads (*P < *0.05). The duodenum revealed significantly lower viral loads in the La Sota group (*P < *0.01) than in the rAdv-HN group; all other findings were found to be nonsignificant (Fig. 11A). Multiple comparisons in the spleen revealed that the La Sota group had significantly lower viral loads (*P < *0.001) than the CC, wtAdv, and rAdv-ChGM-CSF groups. Moreover, the rAdv-ChGM-CSF-HN group revealed significantly lower viral loads (*P < *0.01) than the CC and wtAdv groups, as well as (*P < *0.05) than the rAdv-ChGM-CSF group. Multiple comparisons in the bursa of Fabricius revealed that the CC and rAdv-HN groups as well as the La Sota and rAdv-ChGM-CSF-HN groups had significantly lower viral loads (*P < *0.05, *P < *0.01, and *P < *0.001, respectively) than the rAdv-ChGM-CSF group (Fig. 11A). In addition, as can be seen in Fig. 11B, when comparing the viral loads of the challenged chickens per group to one another, those of the La Sota and rAdv-ChGM-CSF-HN groups had significantly lower viral loads (*P < *0.001) than those of the CC, wtAdv, and rAd-ChGM-CSF groups, whereas the viral loads of the rAdv-HN were considerably lower (*P < *0.05) than those of the CC and wtAdv groups, as well as (*P < *0.01) than the rAd-ChGM-CSF group.

**FIG 12:**
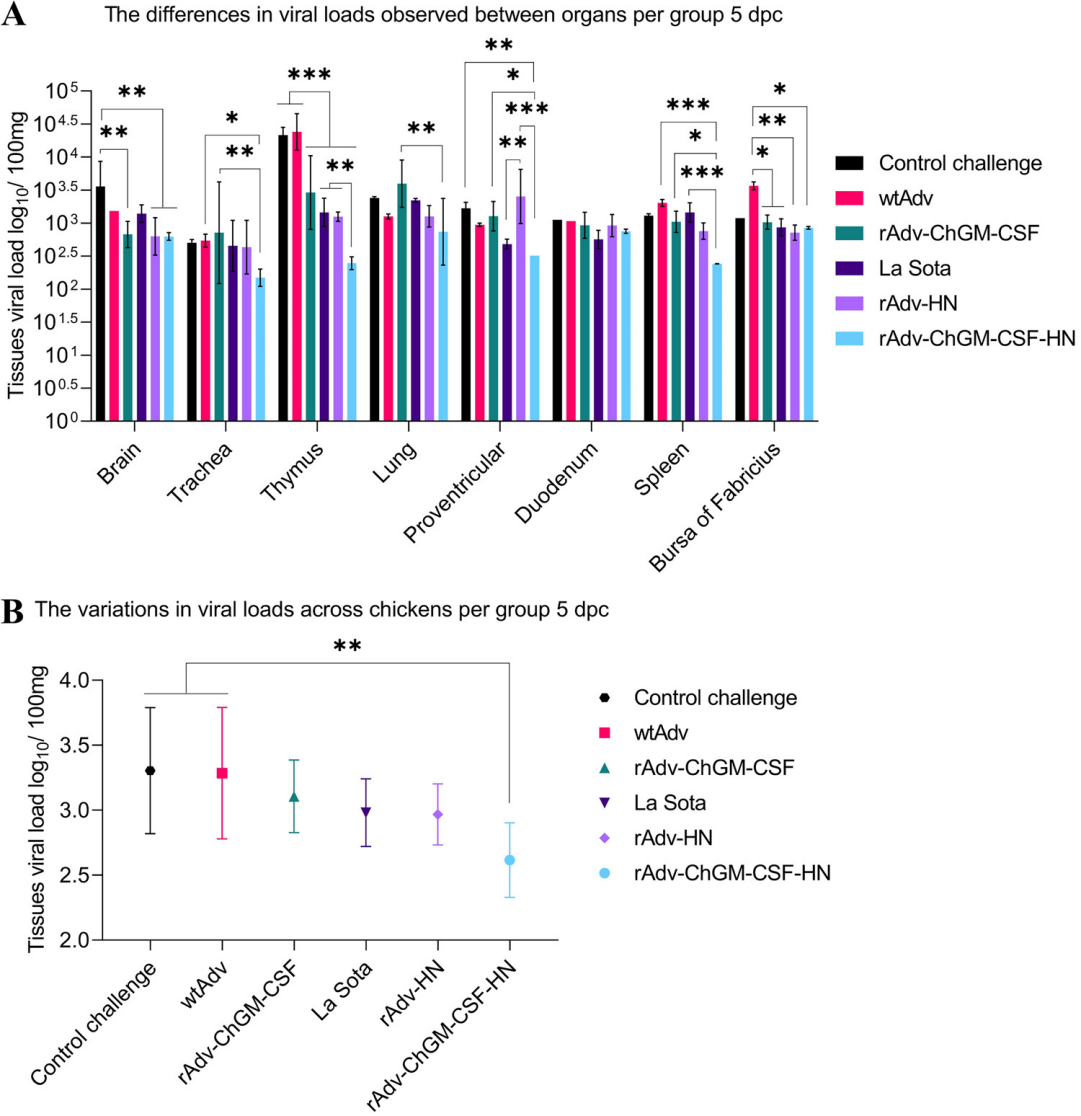
At 5 dpc, the NP gene of the vNDV C22-strain was targeted with specific primers for SYBR green qPCR, which assessed viral levels in the various organs, and the results were evaluated against the standard curve that was *y* = 3.591 and *x* = 41.544, with correlation coefficients (*R*^2^) of 0.999. (A) Differences in viral loads observed among organs per group. (B) Variations in viral loads across chickens per group. Each bar indicates the mean ± one standard deviation. The statistically significant differences are indicated by asterisks (*, *P < *0.05; **, *P < *0.01; ***, *P < *0.001).

As shown in Fig. 12A, the brains of the rAdv-ChGM-CSF, rAdv-HN, and rAdv-ChGM-CSF-HN groups revealed significantly lower tissue viral loads (*P < *0.01) than the CC group at 5 dpc. In the trachea, the rAdv-ChGM-CSF-HN group had significantly lower viral loads (*P < *0.05 and *P < *0.01) compared to the wtAdv and rAdv-ChGM-CSF groups, respectively. In the thymus, the rAdv-ChGM-CSF, La Sota, rAdv-HN, and rAdv-ChGM-CSF-HN groups were found to have significantly lower viral loads (*P < *0.001) than the CC and wtAdv groups, while the rAdv-ChGM-CSF-HN group revealed significantly lower viral loads (*P < *0.01) in comparison with the La Sota and rAdv-HN groups. In the lung, it was found that the rAdv-ChGM-CSF-HN group had significantly lower viral loads (*P < *0.01) than the rAdv-ChGM-CSF group. However, all other findings were nonsignificant (Fig. 12A). Additionally, in the proventriculus, the La Sota group had significantly lower viral loads (*P < *0.01) than the rAdv-HN group. Also, the rAdv-ChGM-CSF-HN group had significantly lower viral loads (*P < *0.05, *P < *0.01, and *P < *0.001, respectively) than the rAdv-ChGM-CSF, CC, and rAdv-HN groups, but the results were varied. In the duodenum, there was no statistically significant difference between the treatments. In the spleen, the rAdv-ChGM-CSF-HN group had significantly lower viral loads (*P < *0.001 and *P < *0.05, respectively) than the wtAdv and La Sota groups, as well as the Adv-ChGM-CSF group. In the bursa of Fabricius, the rAdv-ChGM-CSF, La Sota, and rAdv-ChGM-CSF-HN groups were found to have significantly lower viral loads (*P < *0.05) than the wtAdv group, but the rAdv-HN group revealed significantly lower viral loads (*P < *0.01) than the wtAdv group (Fig. 12A). In contrast, as shown in Fig. 12B, the tissues from the rAdv-ChGM-CSF-HN group exhibited the lowest viral loads (*P < *0.01), particularly compared to the CC and wtAdv groups. This indicates that the ChGM-CSF and HN epitopes combination (rAdv-ChGM-CSF-HN) significantly reduces the viral loads in various tissue types when faced with a challenge, suggesting that it may have potential antiviral efficacy through preventing viral replication.

### Viral shedding.

As shown in Table 1, with the exception of the La Sota group, all other challenged groups were found to be shedding viruses through the oropharynx. However, the levels of viral shedding in the control groups CC and wtAdv (5/5 chickens) were higher than those in the rAdv-ChGM-CSF group (4/5 and 2/5) at 2 dpc and 4 dpc, respectively. Moreover, the rAdv-HN group had lower viral shedding (3/5 and 2/5) at 2 dpc and 4 dpc compared to the rAdv-ChGM-CSF-HN group, which had 3/5, 3/5, and 1/5 at 2 dpc, 4 dpc, and 6 dpc. As presented in Table 1, cloacal viral shedding was observed in all groups except for the La Sota group. Substantial viral shedding was detected in the control groups, particularly in the CC and wtAdv groups (5/5 and 5/5), at 2 dpc and 4 dpc compared to the rAdv-ChGM-CSF group (3/5, 3/5, and 2/2) at 2 dpc, 4 dpc, and 6 dpc, respectively. Interestingly, viral shedding in the rAdv-ChGM-CSF-HN group was very low, with only 1/5 at 4 dpc, compared with the rAdv-HN group with 1/5, 1/5, 2/5, and 1/5 at 2 dpc, 4 dpc, 6 dpc, and 8 dpc, respectively. It has been demonstrated that ChGM-CSF, in combination with the HN epitope (rAdv-ChGM-CSF-HN), plays a vital role in shortening the duration of viral shedding by inhibiting viral replication.

**TABLE 1:** Chickens that shed viruses throughout the oropharynx and cloaca

Group	Data from time point^*a*^^,^^*b*^:						
	2 dpc	4 dpc	6 dpc	8 dpc	10 dpc	12 dpc	14 dpc
Oropharynx							
Control challenge	5/5	5/5	–	–	–	–	–
wtAdv	5/5	5/5	–	–	–	–	–
rAdv-ChGM-CSF	4/5	2/5	0/2	–	–	–	–
La Sota	0/5	0/5	0/5	0/5	0/5	0/5	0/5
rAdv-HN	3/5	2/5	0/5	0/5	0/5	0/5	0/5
rAdv-ChGM-CSF-HN	3/5	3/5	1/5	0/5	0/5	0/5	0/5
Cloca							
Control challenge	5/5	5/5	–	–	–	–	–
wtAdv	5/5	5/5	–	–	–	–	–
rAdv-ChGM-CSF	3/5	3/5	2/2	–	–	–	–
La Sota	0/5	0/5	0/5	0/5	0/5	0/5	0/5
rAdv-HN	1/5	1/5	2/5	1/5	0/5	0/5	0/5
rAdv-ChGM-CSF-HN	0/5	1/5	0/5	0/5	0/5	0/5	0/5

a Number of the positive swab samples/total number of chickens examined in each time point.

b –, No survival.

## DISCUSSION

Several studies have shown that adjuvant cytokines are capable of enhancing the immune system’s ability to function. Because of their instability in nature, cytokine molecules have not been used as adjuvants in clinical or field applications. In our current project, to avoid the short half-life of both cytokines and antigenic determinants, the ChGM-CSF and the HN epitopes of the virulent NDV genotype VII-C22 strain were delivered separately or simultaneously through expression of the genes incorporated into the adenovirus 5 genome, rather than by directly adding the protein into the vaccine. As long as the recombinant adenovirus is active, the ChGM-CSF and the HN epitopes can be expressed consistently and cumulatively. This strategy is also preferable because it reduces the risk of immunodeficiency induced by high doses of cytokines given in high concentrations for a short period of time (59). In this study, the novel viruses were found to be thermostable when they were exposed to the various temperatures, as shown in Fig. 4B. In terms of stability, via recombination and vegetative viral passage, adenovirus can sustain infectivity for long periods of time, even at high temperatures (60–62). In this study, we used an adenoviral delivery system to simultaneously express the ChGM-CSF and vNDV C22-HN epitope instead of a live attenuated viral vaccine or an inactivated vaccine to avoid the problems of genotype mismatch, viral revolution, reservoir, MDAs, and insufficient induction of humoral immune response. In addition, as can be seen in Fig. 5, the potential vaccines’ immune responses were evaluated in DF1 cells, and the results showed that they functioned very well *in vitro*. Therefore, with rAdv-ChGM-CSF-HN as a live recombinant NDV transgenic vaccine, the humoral immune response improved and potentially worked, and NDV-specific antibodies were found to be significantly elicited at high levels with persistence against NDV compared with rAdv-HN and La Sota, both before and after the challenge test (Fig. 6) *in vivo*. According to recent studies, using ChGM-CSF as a bio-adjuvant enhanced the kinetics and quantity of the chicken antibody immunological response (1, 55–58). The spread of ND can be prevented through immunizations, proper management, and biosecurity. However, vaccination failure persists despite the same serotype due to an antigenic mismatch between vaccines and field strains (30–35). Additionally, MDAs in chickens have the potential to reduce vaccination efficacy by neutralizing the vaccine and, therefore, its potency (41, 42). Furthermore, inoculating chickens with inactivated vaccines resulted in an atypical ND immune response and/or an inadequate humoral immune response (44, 45). We found that when the ChGM-CSF and the HN epitopes were simultaneously expressed (rAdv-ChGM-CSF-HN), immunological gene expression of IFN-α, IFN-β, and IFN-γ, as well as IL-1β, IL-2, IL-16, IL-18, and IL-22, was enhanced, and cytokine instability was reduced, indicating that with the aid of ChGM-CSF, the humoral and adaptive immune system was in harmony because of especially high levels of IFN-γ and IL- 18, as well as others cytokines (Fig. 7). The type 1 interferons (IFNs) IFN-α and IFN-β, have antiviral effects and can postpone the onset and severity of viral diseases (63). Upregulation of type 2 interferon, IFN-γ, in chicken spleens has antiviral properties and stimulates macrophages and natural killer cells (64), including class I and class II major histocompatibility complex (MHC) proteins (65). In addition, IFN-γ coadministration with the ND DNA vaccine increased the cell-mediated immune response to NDV (66). Also, GM-CSF plays an important role in the recruitment and maturation of dendritic cells (DCs) (67), which play a role in the activation of T cell immune responses as well as the production of B cells (68, 69). However, the lentogenic La Sota strain induced a phenotypic maturation of immature DCs (imDCs), resulting in a reduction in T cell responses (70). Our findings indicated that when ChGM-CSF was used, the humoral and cell-mediated immune responses performed well, as evidenced by significantly higher levels of IL-18 and markedly elevated serum antibody (HI) titers, and elicited Th1-IFN-γ in the rAdv-ChGM-CSF-HN group compared to the La Sota and rAdv-HN groups. Previous results support our findings that ChIL-18 increased Th1-IFN-γ and Th2-IL-4 secretion, T and B lymphocyte proliferation, HI antibody titers, and ratios of CD4^+^ to CD8^+^ in chickens (71, 72). Also, a single dose of ChGM-CSF fused or combined with spike glycoprotein (S1) of infectious bronchitis virus (IBV) *in ovo* vaccination boosted humoral and cellular immunity (58); in addition, the chickens in the rClone30-chGM-CSF-immunized group had higher CD4^+^ and CD8^+^ T cell proliferative responses (56), and following a booster immunization with chicken ChGM-CSF, both Th1 and Th2 cytokines were increased (1, 55). We also investigated which vaccines protected chickens against challenge, and the results revealed that 100% of the CC, wtAdv, and rAdv-ChGM-CSF groups developed severe NDV-specific clinical indications and viral shedding and died within 7 days. However, the rAdv-ChGM-CSF group had a delayed onset of clinical manifestations, viral shedding, and mortality. None of the three vaccinated groups (La Sota, rAdv-HN, and rAdv-ChGM-CSF-HN) showed clinical signs or died (Fig. 8). Furthermore, the rAdv-HN and rAdv-ChGM-CSF-HN groups both shed viruses, but the La Sota group did not. However, rAdv-ChGM-CSF-HN rapidly controlled and stopped viral shedding, suggesting the potential antiviral efficacy of ChGM-CSF in improving humoral and cellular immunity; therefore, the virulent Newcastle disease virus C22 strain pathological lesions are completely prevented postchallenge in the rAdv-ChGM-CSF-HN group than those in the La Sota and rAdv-HN groups (Fig. 9 and 10). According to previous studies, the NDV vaccine with rChGM-CSF bio-adjuvant showed fewer lesions and less mortality and viral shedding than the vaccine alone (1, 55, 57, 58). In the present study, unvaccinated groups had significantly higher viral loads than vaccinated groups at 3 dpc, whereas the La Sota and rAdv-ChGM-CSF-HN groups had significantly lower viral loads than the rAdv-HN group (Fig. 11). At 5 dpc, the unvaccinated groups, namely, CC and wtAdv, had higher viral loads than the rAdv-ChGM-CSF and vaccinated groups, while the rAdv-ChGM-CSF-HN group had the lowest viral loads compared to the La Sota and rAdv-HN groups, as well as all other challenged groups. Due to its potential antiviral activity, ChGM-CSF, alone or along with the HN epitope, dramatically reduces viral replication in tissues (Fig. 12). Previous studies support our findings because when the ChGM-CSF was used as a biological adjuvant, the viral loads were reduced (1, 55). Importantly, the La Sota strain suppressed T cell responses by maturing immature DCs, which regulate innate and adaptive immunity (70). Moreover, the interferon antagonistic activity of the NDV V protein and/or inhibition of apoptosis in target cells promotes viral replication (18, 73–76). As a result, current live attenuated NDV vaccines have limited effects on viral replication in vaccinated flocks and can serve as reservoirs; even so, increased virulence following passage via chicken air sac evolution of the quasispecies diversity of NDV could be a risk factor for future velogenic NDV outbreaks (7, 21, 36–38, 40). Interestingly, the rAdv-ChGM-CSF-HN was found to significantly improve the innate and adaptive immunological conditions of the vaccinated chickens, bridging the knowledge gap between vaccine manufacturing and application; therefore, NDV eradication and improved poultry health can be achieved using field-circulating viruses, biological adjuvants, and transgenic recombinant live vaccines.

**Conclusion.** The promising vaccine rAdv-ChGM-CSF-HN (adenoviral simultaneously expressed ChGM-CSF with vNDV C22-strain HN epitope) provided significant antiviral activity in both humoral and cellular immunity with persistent, measurable, and long-lasting immunity, as well as increased protective immune efficacy in the face of viral challenge. Consequently, ChGM-CSF, which serves as a bio-adjuvant in combination with the epitope of the field-circulating NDV strain via a live adenoviral expression delivery system, can be used to create a safe, genotype-matched, and promising universal recombinant vaccine that has the potential to slow or stop the NDV evolutionary process, eradicate disease, and improve poultry health and hence the economy.

## MATERIALS AND METHODS

### Ethics statement.

Throughout Northwest A&F University, the Ethics Committee inspected and approved all laboratory and experimental materials, as well as all animal-related procedures. The independent Animal Care and Use Committee in Shaanxi Province, China, issued guidelines that were adhered to and strictly followed.

### Viruses, construction plasmids, cells, and rescuing viruses.

**FIG 13:**
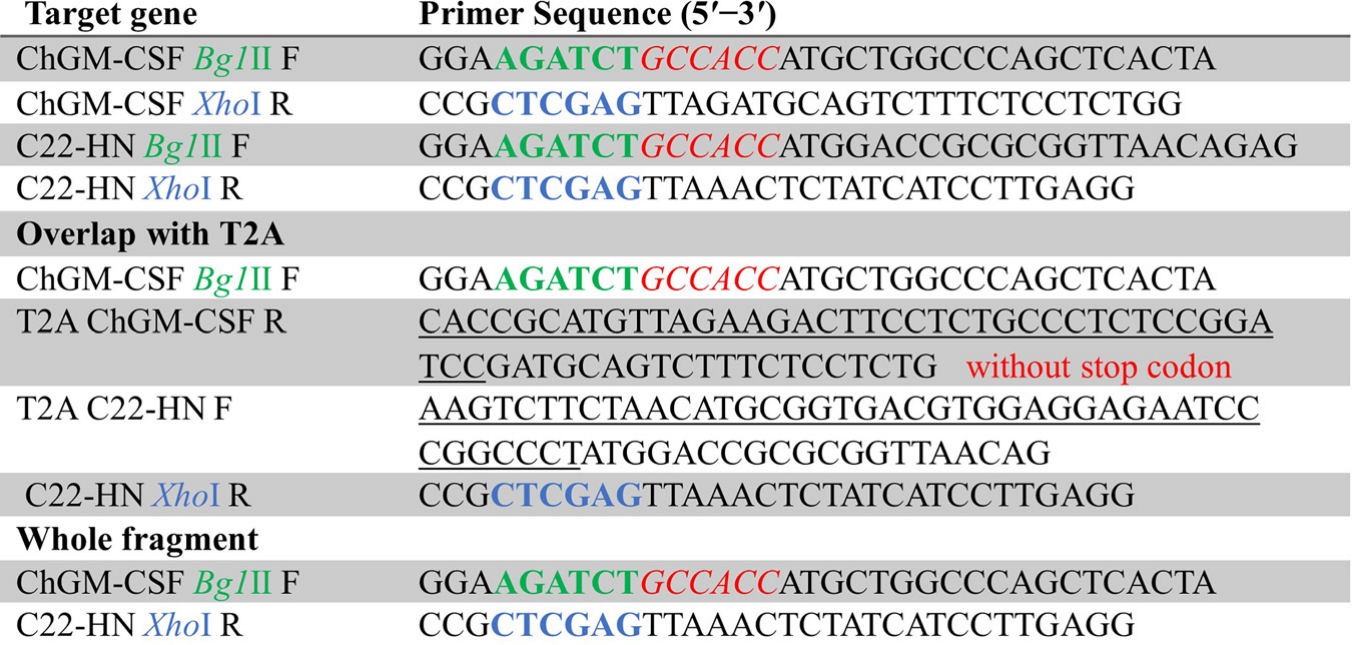
The primers that were used during the PCR-based amplification procedure. The bold green and blue colors signify where the restriction enzyme sequences are, the italic red color shows the Kozak sequence that is used in the forward primers, and the underline indicates where the linker is when the PCR is overlapping.

Both the vNDV genotype VII C22 strain and the La Sota virus strain are maintained in our laboratory, and they were amplified and titrated in 9-day-old embryonated chicken eggs, as well as the wtAdv. Additionally, in accordance with the procedures described previously with some modifications (77, 78), in brief, using specialized designated primers with unique restriction enzyme sites, the Kozak sequence (as shown in Fig. 13) was used for PCR-based amplification to amplify either the ChGM-CSF (accession no. EU939770.1) or the HN epitope of the vNDV genotype VII C22-strain. Moreover, the ChGM-CSF-HN was amplified in the same manner, but adding the self-cleaving peptides, linked from the Thosea asigna virus (T2A) (accession no. OF208254.1), with a Gly-Ser-Gly (GSG) spacer on the N terminus of a 2A peptide helps with the efficiency to generate a multicistronic vector as previously described (79). Then, the open reading frames (ORFs) of the genes of interest (GOI) were introduced as an additional transcription unit into a pAdTrack-CMV vector. The constructions were completed successfully based on the gene sequencing provided by Tsingke Co., Ltd., Xian, China, and the recombination was validated by restriction endonuclease analysis. The newly generated plasmids were then linearized with the restriction endonuclease PmeI before being cotransformed into competent AdEasier cells, which are BJ5183 derivatives with the adenoviral backbone plasmid pAdEasy-1. Overall, the proven recombinant adenovirus plasmids were digested with PacI to release both inverted terminal repeats (ITRs). Following the transfection of 4 μg of the constructed recombinant adenoviral plasmids with Lipofectamine 2000, the potential viruses were then rescued, amplified, and titrated in human embryonic kidney HEK-293A packaging cells (ATCCCRL1573). The HEK-293A cells were cultivated in Dulbecco’s modified Eagle medium (DMEM) supplemented with 2 mM l-glutamine, 10% fetal bovine serum, 100 U/mL penicillin, and 100 μg/mL streptomycin.

### Biological activity of the rescued viruses.

In order to evaluate the relative mRNA gene expression, Western blot analysis, immunofluorescent antibody assay, growth curves, thermostability, and genetic stability of the reused viruses—rAdv-ChGM-CSF, rAdv-HN, and rAdv-ChGM-CSF-HN—were compared with those of wtAdv in HEK-293A cells and chicken embryonic fibroblast cells (DF1). The following tests were performed: The HEK-293A cells were infected with the candidate viruses at a multiplicity of infection (MOI) of 0.01, and after 24 h of infection, total RNA was extracted from the infected cells using TRIzol reagent in accordance with the manufacturer’s instructions (TaKaRa Biomedical Technology Beijing Co., Ltd., China) to determine the relative mRNA gene expression levels. Then, 2 μg of the total extracted RNA was subjected to reverse transcription cDNA synthesis using a cDNA synthesis kit (GenStar Co., Ltd., China) as directed by the manufacturer. Table 2 lists the special designated primers that were used for quantitative PCR (qPCR). In brief, as displayed elsewhere (80), the qPCR analysis was performed in triplicate wells for each specimen in accordance with the manufacturer’s instructions, using a SYBR green (GenStar Co., Ltd.) and a real-time thermocycler detection system. The target genes’ expression levels were compared with the expression of the human β-actine gene (Δ*CT* method treatment cells = threshold cycle [*C_T_*] target – *C_T_* β-actine) and expressed as an *n*-fold increase or decrease relative to the NC control (2^–ΔΔ^*^CT^*, ΔΔ*CT* = Δ*CT* treat-cells – Δ*CT* NC) as mentioned in reference 81.

**TABLE 2:** Primers used in qPCR-based detection

Target gene	Primer	Primer sequence (5′−3′)
ChGM-CSF	Forward	F: ACCTGTCTGCGTAACAACCT
	Reverse	R: CAGCTGAAAGACGATTCCGC
C22-HN	Forward	F: CCCGCTGGCACTACTAAACA
	Reverse	R: GCTATTGTTCTCAGCCCCGT
β-actine	Forward	F: AGACCTGTACGCCAACACAG
	Reverse	R: TTCTGCATCCTGTCGGCAAT

For Western blot analysis, HEK-293A cells were infected with an MOI of 0.01, and 24 h after infection, the proteins of rAdv-HN and rAdv-ChGM-CSF-HN from the infected cells were targeted using a mouse anti-HN primary monoclonal antibody followed by a goat anti-mouse secondary antibody, while the rAdv-ChGM-CSF protein was detected using a rabbit anti-chicken GM-CSF primary polyclonal antibody followed by a goat anti-rabbit secondary antibody. While tubulin was employed as the housekeeping gene, it was also targeted using a mouse anti-tubulin primary antibody and a goat anti-mouse secondary antibody. Also, a mouse anti-HN primary monoclonal antibody and a goat anti-mouse red fluorescent secondary antibody were used to detect the IFA of rAdv-HN and rAdv-ChGM-CSF-HN in the HEK-293A-infected cells at 24 h postinfection, while a rabbit anti-chicken GM-CSF primary polyclonal antibody and a goat anti-rabbit red fluorescent secondary antibody were used to detect the IFA of rAdv-ChGM-CSF and rAdv-ChGM-CSF-HN.

In HEK-293A cells infected with an MOI of 2, the newly generated viruses were examined for growth curves, thermostability, and genetic stability. In the growth process, cells were cultured in 96-well plates, infected using a serial dilution method, and monitored every 6 h for up to 5 days, and the 50% tissue culture infectious dose (TCID_50_) was estimated based on these observations, as described elsewhere (82, 83). Additionally, in order to evaluate the thermostability of the viruses that were generated, an MOI of 2 of the viruses was subjected to a variety of temperature points, including being unexposed or being exposed at 37°C, 42°C, 47°C, and 52°C for 1 h in a water bath. After that, they were serially diluted, cultured in HEK-293A cells in 96-well plates, and monitored for up to 5 days, and finally, the TCID_50_ was calculated. Furthermore, the genetic stability of the newly rescued viruses was tested by passage in HEK-293A cells. The cells were infected for 24 h and then exposed to three cycles of freezing and thawing before being used to infect fresh cells for up to 20 generations. Finally, PCR-based amplification and sequencing analysis were carried out to identify the inserted GOI.

In the DF1 cells infected with an MOI of 2 of the candidate viruses, post-48 h of infection, the relative mRNA expression levels of IFN-α, IFN-β, IFN-γ, IL-1β, IL-2, IL-16, IL-18, and IL-22 were detected; the primers that were utilized for qPCR are listed in Table 3. In addition, the IFA of rAdv-HN and rAdv-ChGM-CSF-HN in the DF1-infected cells at 48 h postinfection was targeted using a mouse anti-HN primary monoclonal antibody and a goat anti-mouse red fluorescent secondary antibody, while the IFAs of rAdv-ChGM-CSF and rAdv-ChGM-CSF-HN were targeted using a rabbit anti-chicken GM-CSF primary polyclonal antibody and a goat anti-rabbit red fluorescent secondary antibody.

**TABLE 3:** Primers used for qPCR

Target gene	Primer	Primer sequence (5′−3′)	Reference or source
IFN-α	Forward	F: GACATGGCTCCCACACTACC	53
	Reverse	R: AGGCGCTGTAATCGTTGTCT	
IFN-β	Forward	F: GCTCACCTCAGCATCAACAA	53
	Reverse	R: GGGTGTTGAGACGTTTGGAT	
IFN-γ	Forward	F: TGAGCCAGATTGTTTCGATG	53
	Reverse	R: CTTGGCCAGGTCCATGATA	
IL-1β	Forward	F: CTGGGCATCAAGGGCTACAA	85
	Reverse	R: CGGTAGAAGATGAAGCGGGT	
IL-2	Forward	F: TTGGCTGTATTTCGGTAGCA	86
	Reverse	R: GTGCACTCCTGGGTCTCAGT	
IL-16	Forward	F: ATTTGAATCTTTGAGTGCTCCG	53
	Reverse	R: TTGGGACAGGAAGATCTATCCAG	
IL-18	Forward	F: TAACAGATCAGGAGGTGAAATCT	53
	Reverse	R: AAAGGCCAAGAACATTCCTTGTT	
IL-22	Forward	F: AGCCCTACATCAGGAATCGC	53
	Reverse	R: CACATCCTCAGCATACGGGT	
NDV (C22) NP	Forward	F: TGCGGTATTCTGCCTTCGGAT	Designed in this study
	Reverse	R: GCACACTGTTAGCAAAACCAT	
28S	Forward	F: GGTATGGGCCCGACGCT	53
	Reverse	R: CCGATGCCGACGCTCAT	

### Embryonated chicken eggs and experimental animals.

Fertilized chicken eggs were used for the titration of the ND viruses and postchallenge viral shedding detection. A total of 126 Newcastle disease virus-free, healthy 3-month-old chickens were purchased from a poultry hatchery. All of the chickens were maintained in separate incubators (biosafety level 3) with free access to food and water.

### Immunization and challenge test in chickens.

A set of 126 NDV-free, healthy 3-month-old chickens were randomly divided into 7 groups (*n *= 18 per group). After 1 week of acclimatization to their new habitat, they were immunized twice, with the initial immunization taking place on day 1 and the second dose 14 days later. In brief, groups 1 and 2 received 0.2 mL of phosphate-buffered saline (PBS), whereas groups 3, 4, 6, and 7 got 0.2 mL of 1 × 10^10^ TCID_50_/0.1 mL of the adenoviruses that had undergone discontinuous sucrose purification, namely, wtAdv, rAdv-ChGM-CSF, rAdv-HN, and rAdv-ChGM-CSF-HN, respectively, intramuscularly (i.m.), in the pectoralis muscle, while group five was given 0.2 mL of 1 × 10^5^ 50% embryonated infective dose (EID_50_)/0.1 mL of La Sota oculo-nasally. Also, three chickens were chosen at random from each of the groups in this study 4 days postimmunization, and they were examined to determine whether the vaccines posed any health risks. A list of the treatments that were considered appropriate for each of the study groups can be found in Table 4. Unless they were in the nonchallenged (NC) group, all chickens were challenged oculo-nasally with 0.2 mL of 1 × 10^6.5^ 50% embryonated lethal dose (ELD_50_)/0.1 mL of vNDV genotype VII C22 strain on day 28 post-primary immunization, that is, on day-14 following the second dose. The Spearman-Kärber method (82, 83) was used to calculate viral titers, TCID_50_, EID_50_, and ELD_50_.

**TABLE 4:** Experimental design

Group	No. of chickens	Primary immunization day 1	Booster shot day 14	Challenged by vNDV C22-strain 1 × 10^6.5^ ELD_50_/dose day 28
1. Negative control	18	PBS	PBS	−
2. Control challenge	18	PBS	PBS	+
3. wtAdv	18	wtAdv	wtAdv	+
4. rAdv-ChGM-CSF	18	rAdv-ChGM-CSF	rAdv-ChGM-CSF	+
5. La Sota	18	La Sota	La Sota	+
6. rAdv-HN	18	rAdv-HN	rAdv-HN	+
7. rAdv-ChGM-CSF-HN	18	rAdv-ChGM-CSF-HN	rAdv-ChGM-CSF-HN	+

### Serological testing.

Following the OIE standard methodology (84), whole-blood samples were obtained from the wing vein under aseptic conditions every 7 days for a total of 63 days after the first immunization for the assessment of hemagglutination inhibition (HI) titers. Serum samples were separated from whole blood, and four units of NDV were used as the antigen, while chicken red blood cells (RBCs) (1%) were prepared from healthy adult roosters and suspended in normal saline. Titers were expressed as their logarithms to the base 2.

### Quantitative PCR (qPCR).

To achieve total RNA extraction from tissues, we used TRIzol reagent according to the manufacturer’s instructions (TaKaRa Biomedical Technology Beijing Co., Ltd., China). Then, 2 μg of total RNA from each sample was reverse transcribed to generate cDNA using a cDNA synthesis kit (GenStar Co., Ltd., China) as directed by the manufacturer. To determine the splenocyte mRNA gene expression levels of IFN-α, IFN-β, IFN-γ, IL-1β, IL-2, IL-16, IL-18, and IL-22 by qPCR, three randomly selected chickens were examined on day 28 after primary immunization, that is, day 14 after the second immunization, using previously reported primers (53, 85, 86) or newly created primer sequences for the NP gene of the NDV genotype VII C22 strain. Table 3 lists the primers that were used. The qPCR was conducted as previously described (80). In brief, the qPCR analysis was performed in triplicate wells for each specimen in accordance with the manufacturer’s instructions, using SYBR green (GenStar Co., Ltd.) and a real-time thermocycler detection system. The target genes’ expression levels were compared with the expression of the chicken 28s gene (Δ*CT* treatment group = *C_T_* target – *C_T_* 28s) and expressed as an *n*-fold increase or decrease relative to the NC control (2^–ΔΔ^*^CT^*, ΔΔ*CT* = Δ*CT* treatment group – Δ*CT* NC) (81).

### Clinical scores and survival rates.

After the challenge test, which was done on day 28 post-primary immunization, the clinical manifestations in chickens were observed and evaluated on a daily basis, that is, morning and evening up to the 15th day, and they were scored according to the following criteria: healthy = 0, conjunctivitis and ruffled feathers = 1, severe conjunctivitis, ruffled feathers, and prostration = 2, death = 3. At the start of the trial, the total scores from all of the surviving chickens in every group were recorded, and the daily median was recorded for every group in order to produce an overall group score at the end of the experiment. Also, daily mortality rates for each group were recorded for each chicken that died from the infection, except for 3 chickens picked at random from the chickens that had survived on the 3rd and 5th days postchallenge (dpc), which were labeled at the time of the challenge for measurements of tissue viral loads and observations of pathological changes.

### Viral shedding and tissue viral loads.

In order to determine whether they had viral shedding, oropharyngeal and cloacal swabs were taken from 5 chickens in each group at 2-day intervals between 2 and 14 dpc (or even from the complete group if the number of chickens was much less than 5). The presence or absence of viruses was determined in accordance with OIE standard guidelines (84). In order to detect and quantify different tissue viral loads postchallenge, a standard curve was generated from constructed plasmid DNA containing the NP gene of the genotype VII NDV C22 strain and was used to evaluate the qPCR test results against the standard curve that were performed on the brain, trachea, thymus, lung, proventricular, duodenum, spleen, and bursa of Fabricius, following the method described in reference 87.

### Pathological changes.

At 5 dpc, we chose 3 surviving chickens from each group at random and euthanized them to observe the organs’ gross lesions. Tissue samples were taken for histological examination, including brain, lung, proventriculus, cecal tonsils, spleen, and bursa of Fabricius. Tissues were fixed with 4% paraformaldehyde in 1× PBS and then embedded in paraffin, and 4-μm tissue sections were stained with hematoxylin and eosin (88). In addition, the histological lesions were scored descriptively across the tissues based on the presence of hemorrhage, congestion, edema, inflammatory cell infiltration, and degeneration and necrosis, in order to evaluate them for significance and consistency across chickens per group, as shown previously (89, 90). The following criteria were utilized in order to assign a severity rating to each organ: 0 = no change, 1 = focal lesion distribution (low severity), 2 = multifocal lesion distribution (moderate severity), and 3 = diffuse lesion distribution (high severity).

### Statistical analysis.

An ordinary one-way analysis of variance (ANOVA), coupled with Tukey’s test in the case of multiple comparisons, was conducted. Significance was assessed at the 5% level. All statistical analyses were performed with the aid of Prism version 8.2.1 (GraphPad software).

### Data availability.

The article contains all of the data that back up the study’s findings.
