# Supplementary material for: Simultaneous Expression of Chicken Granulocyte Monocyte Colony-Stimulating Factor and the Hemagglutinin-Neuraminidase Epitope of the Virulent Newcastle Disease Virus Genotype VII C22 Strain in a Functional Synthetic Recombinant Adenovirus as a Genotype-Matched Vaccine with Potential Antiviral Activity
Source: Microbiol Spectr. 2023 Apr 10;11(3):e04024-22. doi: 10.1128/spectrum.04024-22 (PMC10269747; doi:10.1128/spectrum.04024-22)
Supplement: Supplemental file 1 — Supplemental material. Download spectrum.04024-22-s0001.pdf, PDF file, 0.3 MB [file spectrum.04024-22-s0001.pdf]

Using the standard curve, the viral loads found in the various tissues were calculated

A

No.		Plasmid concentration (ng)	Avogadro number	Plasmid length (nt)	Comparison factor	Mass of 1 base pair	Copy number
1	dsDNA	1046	6.02E+23	6914	1.00E+09	660	1.38E+11
2	dsDNA	104.6	6.02E+23	6914	1.00E+09	660	1.38E+10
3	dsDNA	10.46	6.02E+23	6914	1.00E+09	660	1.38E+09
4	dsDNA	1.046	6.02E+23	6914	1.00E+09	660	1.38E+08
5	dsDNA	0.1046	6.02E+23	6914	1.00E+09	660	1.38E+07
6	dsDNA	0.01046	6.02E+23	6914	1.00E+09	660	1.38E+06
7	dsDNA	0.001046	6.02E+23	6914	1.00E+09	660	1.38E+05
8	dsDNA	0.0001046	6.02E+23	6914	1.00E+09	660	1.38E+04
9	dsDNA	0.00001046	6.02E+23	6914	1.00E+09	660	1.38E+03
10	dsDNA	0.000001046	6.02E+23	6914	1.00E+09	660	1.38E+02
11	dsDNA	1.046E-07	6.02E+23	6914	1.00E+09	660	1.38E+01
		Number of copies = (Amount of DNA (ng)x (Avogadro number)/ (length of DNA (bp)x1.0E+09X660)					

B

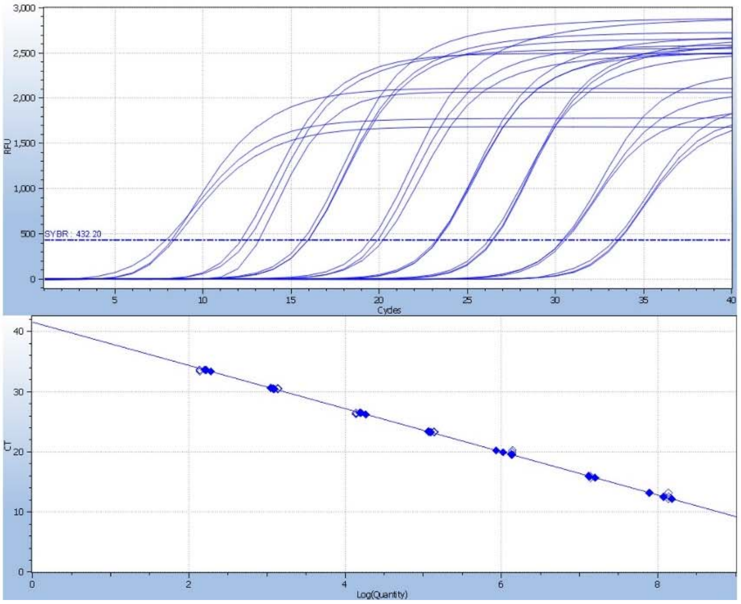

S. 1 Explained how the copy number was computed in (**A**), which led to the creation of the standard curve, which was y = 3.591 x = 41.544 and had a correlation coefficient (R^2) of 0.999 (**B**).
